# The human olfactory system in two proteinopathies: Alzheimer’s and Parkinson’s diseases

**DOI:** 10.1186/s40035-020-00200-7

**Published:** 2020-06-03

**Authors:** Isabel Ubeda-Bañon, Daniel Saiz-Sanchez, Alicia Flores-Cuadrado, Ernesto Rioja-Corroto, Melania Gonzalez-Rodriguez, Sandra Villar-Conde, Veronica Astillero-Lopez, Juan Pablo Cabello-de la Rosa, Maria Jose Gallardo-Alcañiz, Julia Vaamonde-Gamo, Fernanda Relea-Calatayud, Lucia Gonzalez-Lopez, Alicia Mohedano-Moriano, Alberto Rabano, Alino Martinez-Marcos

**Affiliations:** 1 Neuroplasticity and Neurodegeneration Laboratory, Ciudad Real Medical School, CRIB, University of Castilla-La Mancha, 13005 Ciudad Real, Spain; 2 Neurology Service, Ciudad Real General University Hospital, 13005 Ciudad Real, Spain; 3 Pathology Service, Ciudad Real General University Hospital, 13005 Ciudad Real, Spain; 4 Faculty of Health Sciences, University of Castilla-La Mancha, Talavera de la Reina, Spain; 5 Neuropathology Department and Tissue Bank, CIEN Foundation, Carlos III Health Institute, Madrid, Spain

**Keywords:** α-Synuclein, Amyloid-β, Anterior olfactory nucleus, hyposmia, Tau protein

## Abstract

Alzheimer’s and Parkinson’s diseases are the most prevalent neurodegenerative disorders. Their etiologies are idiopathic, and treatments are symptomatic and orientated towards cognitive or motor deficits. Neuropathologically, both are proteinopathies with pathological aggregates (plaques of amyloid-β peptide and neurofibrillary tangles of tau protein in Alzheimer’s disease, and Lewy bodies mostly composed of α-synuclein in Parkinson’s disease). These deposits appear in the nervous system in a predictable and accumulative sequence with six neuropathological stages. Both disorders present a long prodromal period, characterized by preclinical signs including hyposmia. Interestingly, the olfactory system, particularly the anterior olfactory nucleus, is initially and preferentially affected by the pathology. Cerebral atrophy revealed by magnetic resonance imaging must be complemented by histological analyses to ascertain whether neuronal and/or glial loss or neuropil remodeling are responsible for volumetric changes. It has been proposed that these proteinopathies could act in a prion-like manner in which a misfolded protein would be able to force native proteins into pathogenic folding (seeding), which then propagates through neurons and glia (spreading). Existing data have been examined to establish why some neuronal populations are vulnerable while others are resistant to pathology and to what extent glia prevent and/or facilitate proteinopathy spreading. Connectomic approaches reveal a number of hubs in the olfactory system (anterior olfactory nucleus, olfactory entorhinal cortex and cortical amygdala) that are key interconnectors with the main hubs (the entorhinal–hippocampal–cortical and amygdala–dorsal motor vagal nucleus) of network dysfunction in Alzheimer’s and Parkinson’s diseases.

## Background

**Fig. 1:**
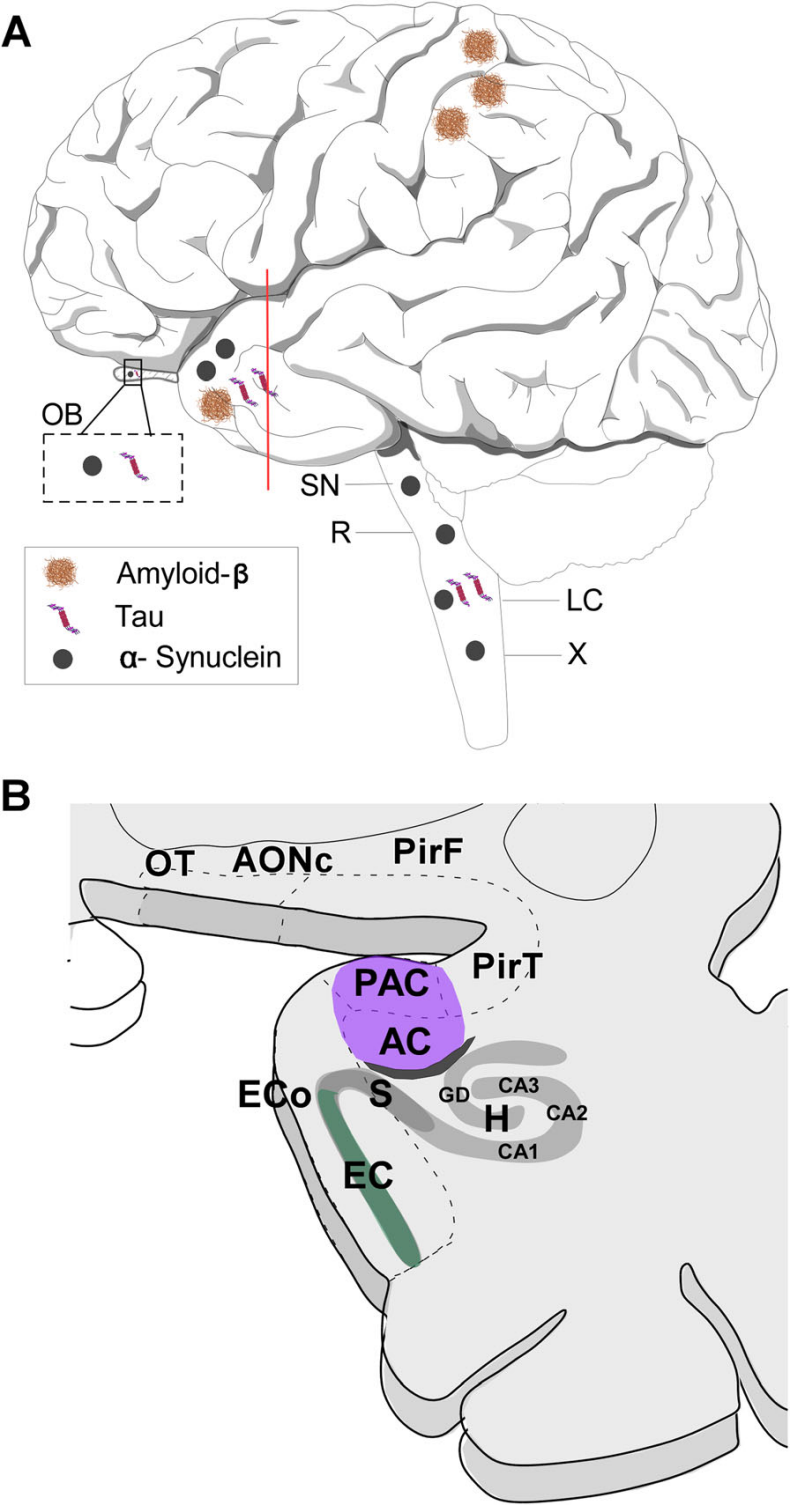
Scheme of the lateral view of the human brain showing the main locations of amyloid-β peptide, tau protein and α-synuclein protein during Alzheimer’s and Parkinson’s diseases (**a**) as well as of a coronal section of the frontal and temporal lobes showing the approximate location of some neuroanatomical structures analysed (**b**). Note that different rostro-caudal levels have been collapsed in order to show all structures of interest. For abbreviations, see list

**Fig. 2:**
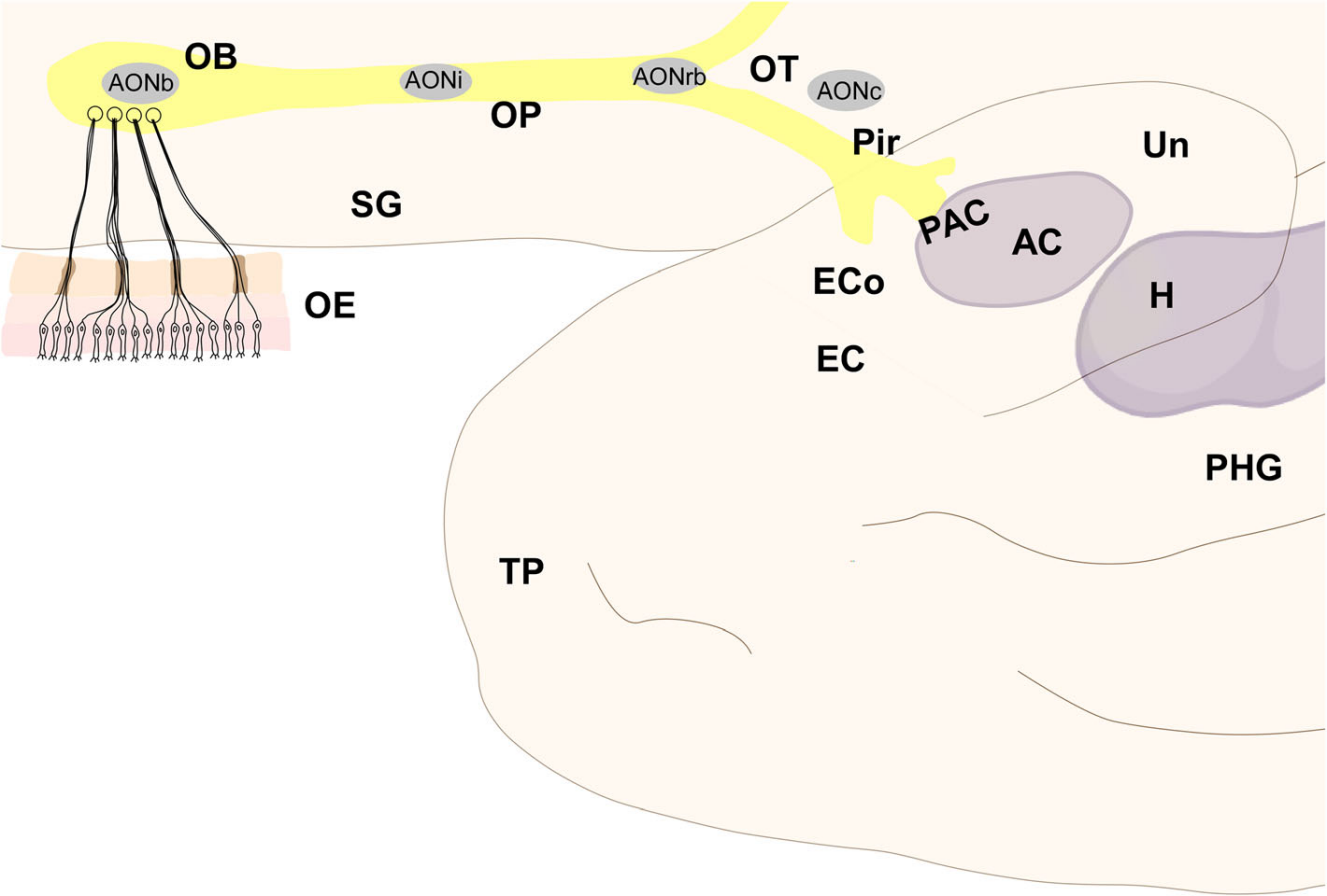
Scheme of the inferolateral view of the frontal and temporal human lobes showing the main components of the olfactory system. Note that olfactory structures are actually located in the medial temporal lobe and visualized making the temporal lobe transparent. For abbreviations, see list

Alzheimer’s [1] and Parkinson’s [2] are the first and second most prevalent neurodegenerative diseases, respectively. The etiology, symptomatology and treatment of these diseases are different. However, they share features that have opened new research strategies. First, hyposmia is an early preclinical symptom [3], and the olfactory system is involved due to pathological aggregates in the initial stages [4] (Figs. 1 and 2). Second, several studies have pointed out that associated proteinopathies could act in a prion-like manner, allowing misfolded proteins to induce further misfolding of native proteins and thus propagate through the nervous system [5]. Third, accumulating evidence points towards glia (microglia and astroglia) as key players in these seeding and spreading mechanisms [6]. The present review aims to update the current knowledge on this topic, including data on patients and postmortem tissue as well as experimental models, and advances the hypothesis that certain structures within the olfactory system constitute “hubs” for connectomic propagation [7] of these proteinopathies.

## Main text

### The human olfactory system

Amongst the human neuroanatomical classifications, only a few consider, based on phylogenetic and overall ontogenetic data, that the olfactory system is formed by three subsystems: olfactory proper, vomeronasal and terminal [8]. However, it is true that the latter two can only be observed in embryos and fetuses [9], and that the olfactory system is comparatively reduced and the vomeronasal system is vestigial in hominids [10].

The human olfactory system [11] includes the olfactory epithelium, the olfactory bulb and the olfactory cortex (Fig. 2) [12–14]. Primary projections go from the olfactory epithelium to the olfactory bulbs, secondary projections go from the olfactory bulb to the olfactory cortex, and tertiary projections mainly go from the olfactory cortex to other structures within the olfactory system and beyond to the amygdaloid complex, the hippocampal formation and the ventral striatum [14]. This scheme, however, is more complex when centrifugal [15] and contralateral [16] connections of the system are considered.

**Fig. 3:**
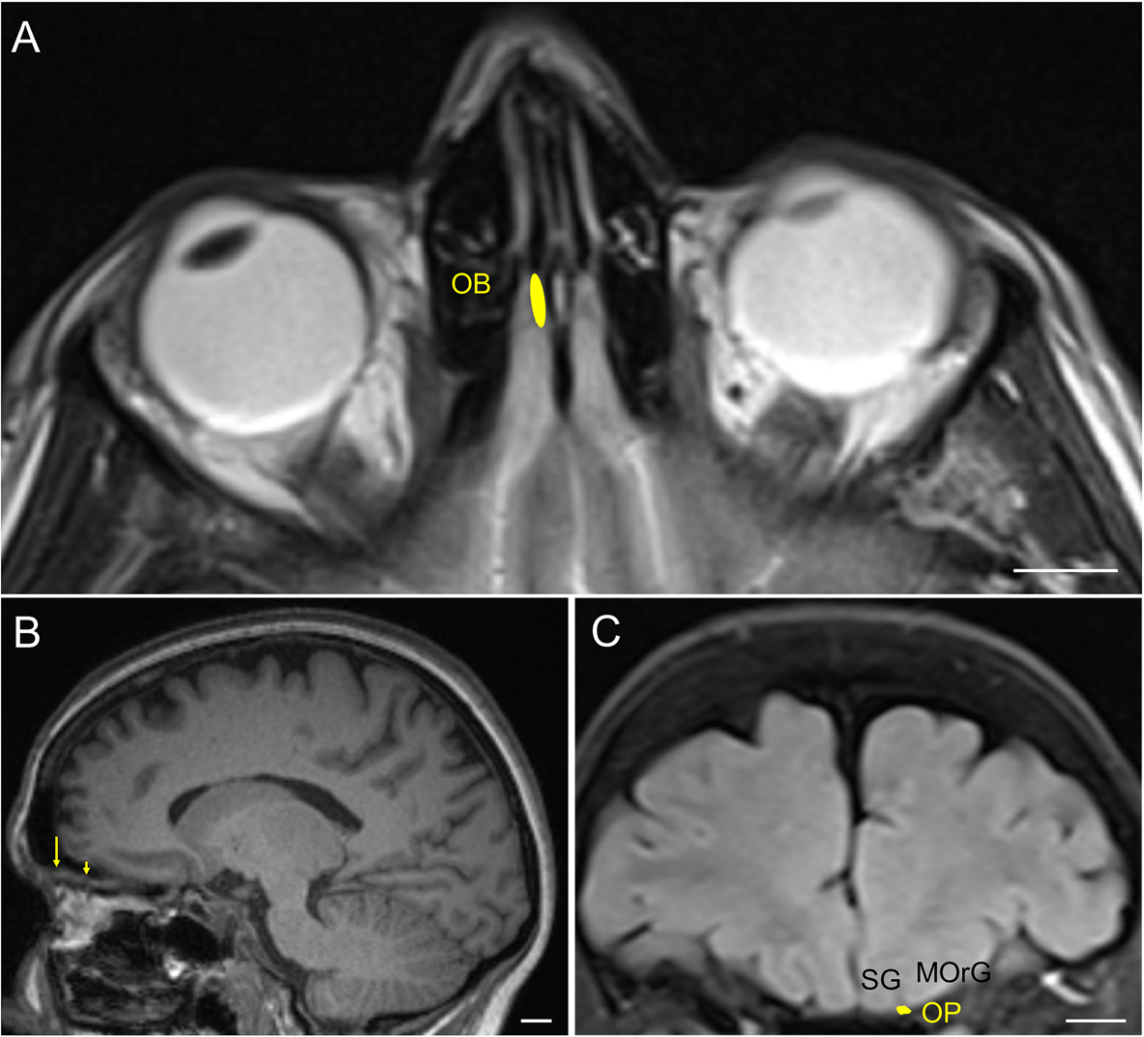
Magnetic resonance images of the human brain illustrating the localisation of the olfactory bulb and olfactory peduncle. **a**: T2 image in an axial plane; **b**: T1 image in a parasagittal plane (arrow points to the olfactory bulb and arrowhead points to the olfactory peduncle); **c**: Coronal FLAIR image in the coronal plane. Calibration bar: 10,000 μm. For abbreviations, see list

**Fig. 4:**
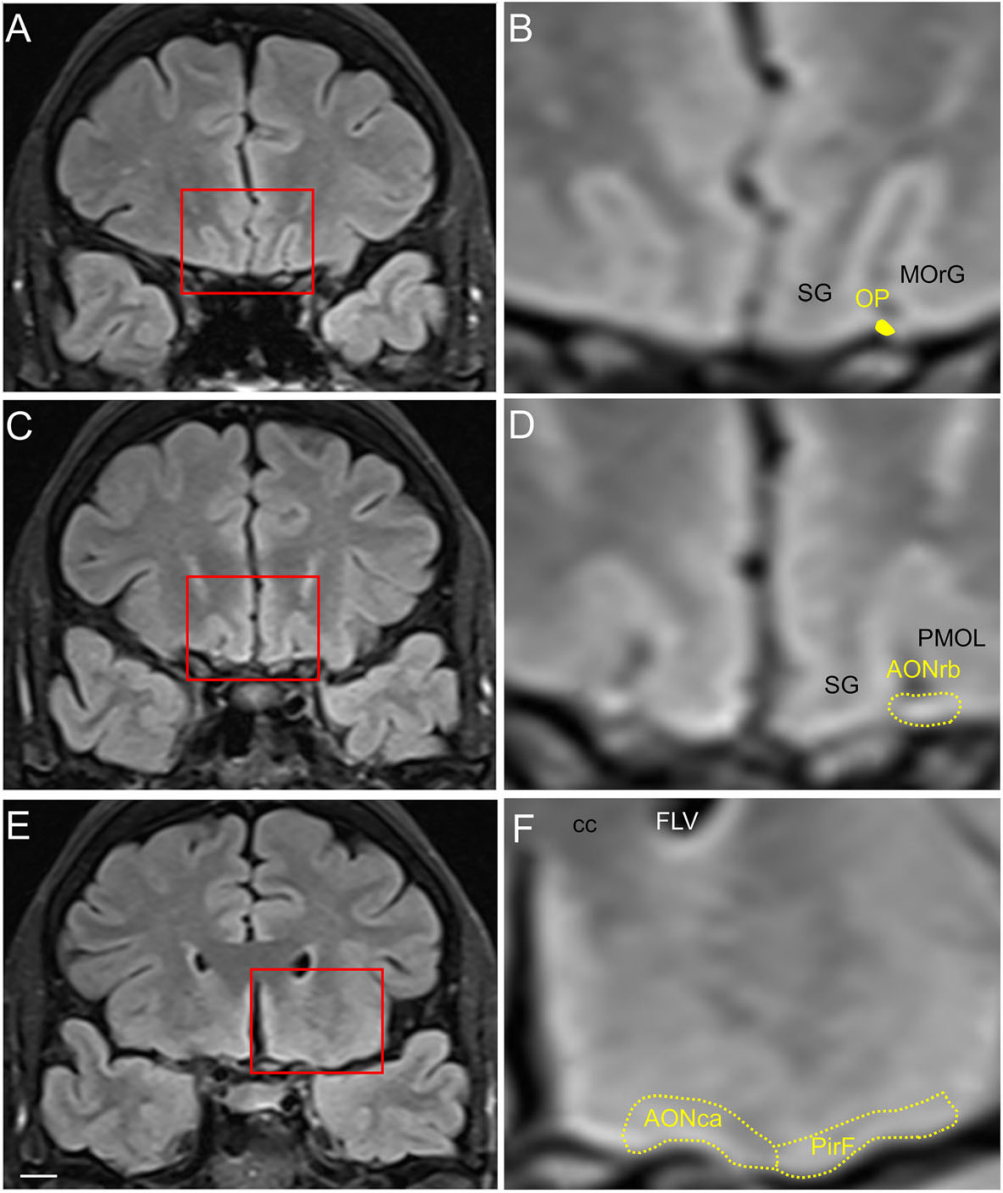
Magnetic resonance images of the human brain illustrating the localisation of rostral olfactory structures. Images correspond to a coronal FLAIR sequence in the coronal plane. Calibration bar: 10,000 μm for (**a**, **c**, **e**) and 2600 μm for (**b**, **d**, **f**). For abbreviations, see list

**Fig. 5:**
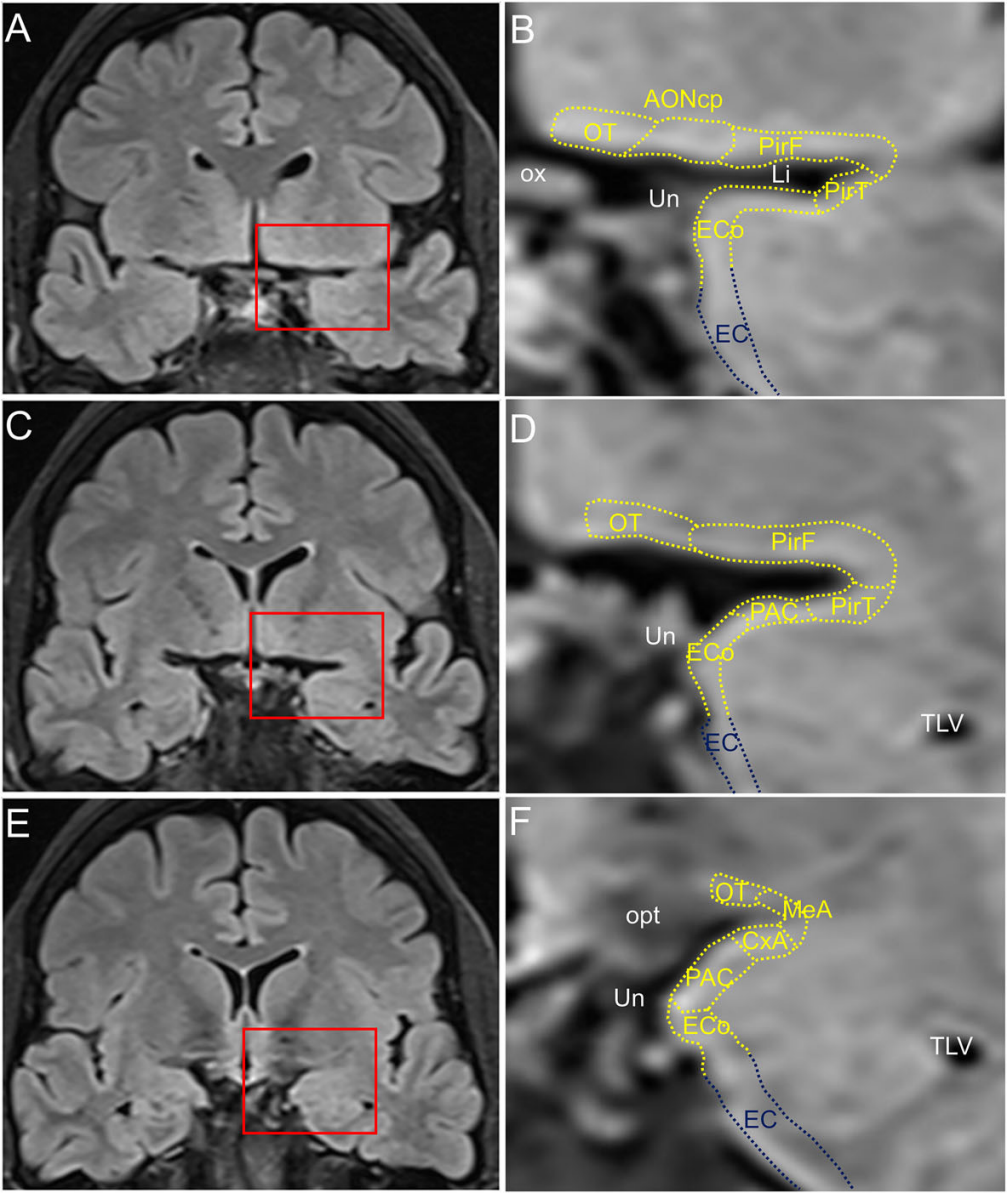
Magnetic resonance images of the human brain illustrating the localisation of caudal olfactory structures. Images correspond to a coronal FLAIR sequence in the coronal plane. Calibration bar: 10,000 μm for (**a**, **c**, **e**) and 2600 μm for (**b**, **d**, **f)**. For abbreviations, see list

**Fig. 6:**
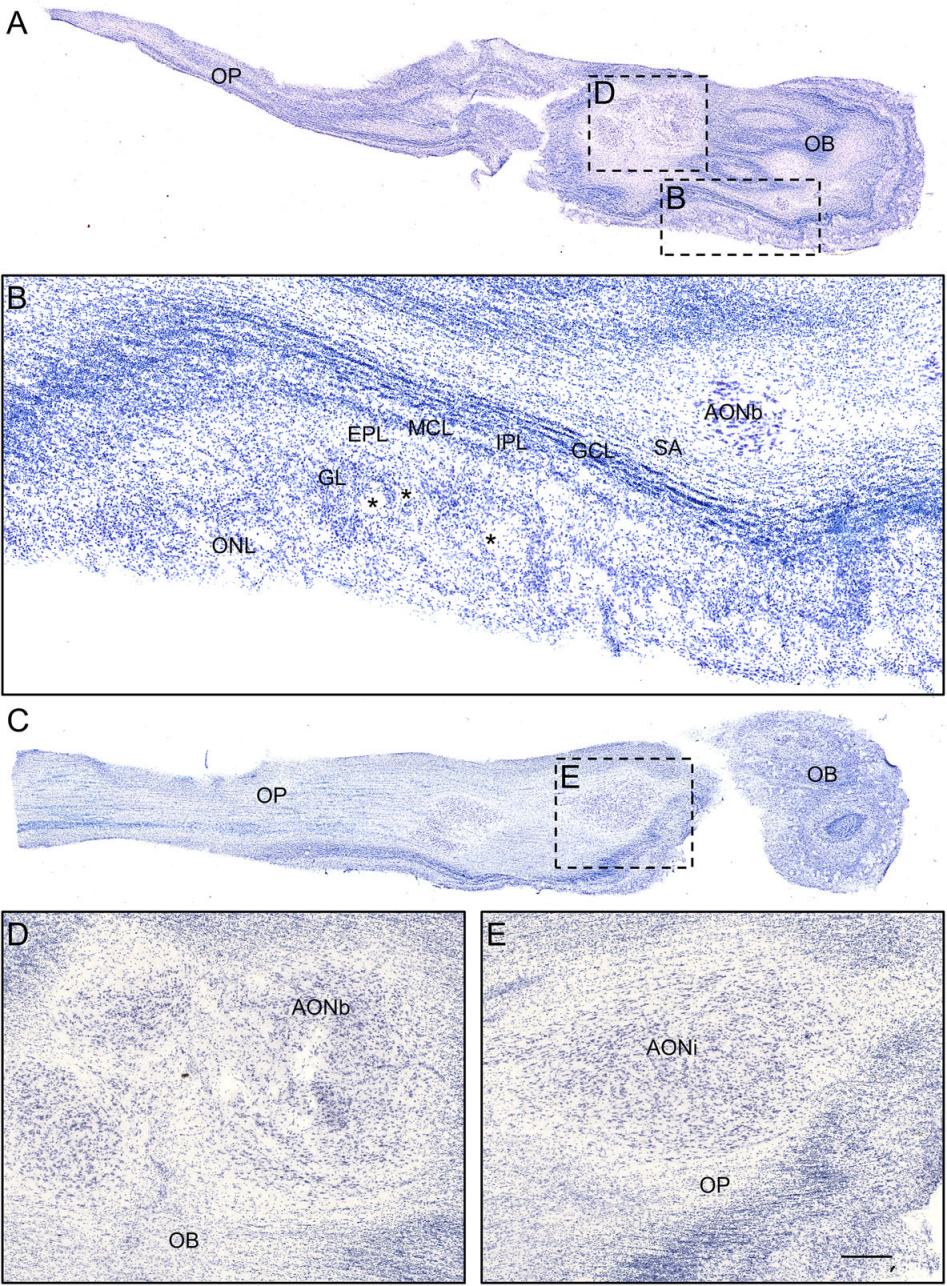
Nissl-stained axial mosaic-reconstructed sections of the human olfactory bulb (**a**) including the different layers (**b**) as well as the olfactory peduncle (**c**). High-power magnifications from framed areas in **a** and **c** are illustrated in **b**, **d** and **e**. Calibration bar: 1000 μm for **a**, **c**, 200 μm for **b**, 100 μm for **d**, **e**. For abbreviations, see list

**Fig. 7:**
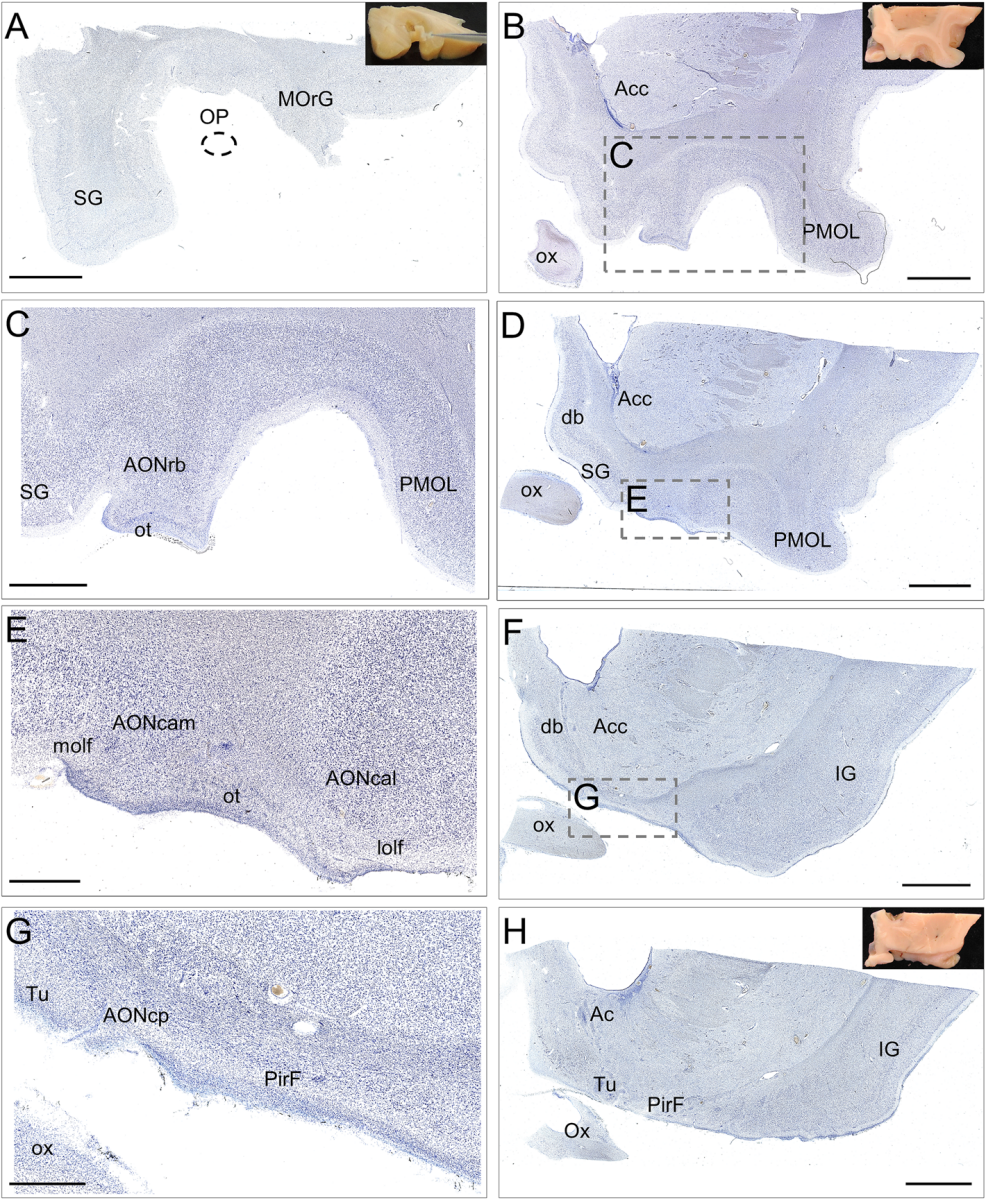
Nissl-stained coronal mosaic-reconstructed sections of the human frontal lobe illustrating olfactory structures (**a**, **b**, **d**, **f**, **h**). Panels **a**, **b** and **h** include pictures of tissue blocks from where sections were obtained. Dashed line in **a** points to the approximate location of the olfactory peduncle. High-power magnifications from framed areas in **b**, **d** and **f** are illustrated in **c**, **e** and **g**, respectively. Calibration bar: 5000 μm for **a**, **b**, **d**, **f**, **h**, 2500 μm for **c**, 1250 μm for **e**, 1000 μm for **g**. For abbreviations, see list

**Fig. 8:**
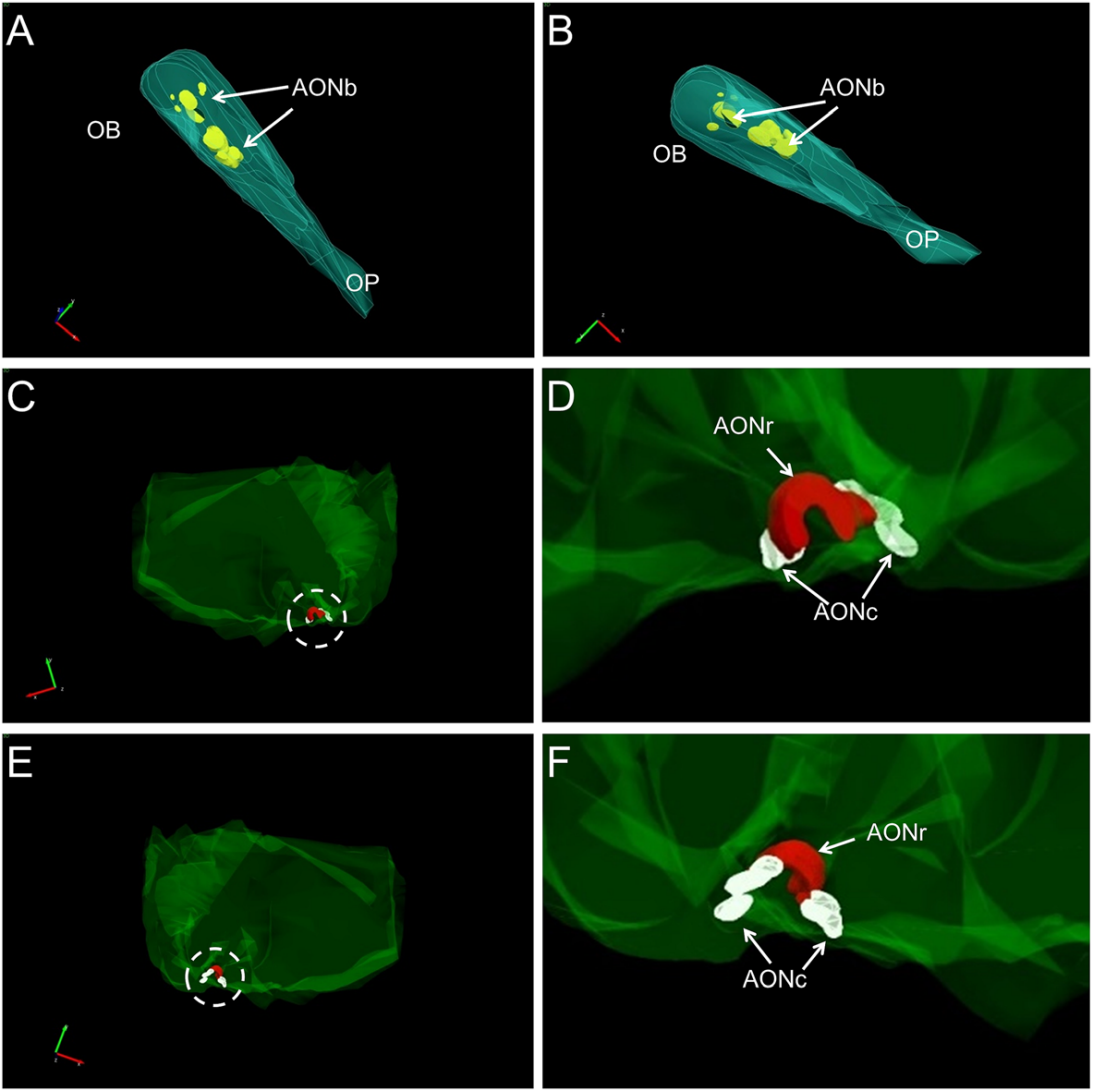
Three-dimensional reconstructions (StereoInvestigator® software from Micro Bright Field Bioscience) starting sections from the human olfactory bulb (**a**, **b**) and frontal lobe (**c**, **e**) illustrating different portions of the anterior olfactory nucleus. High-power magnifications of areas indicated by dashed lines in **c** and **e** are shown in D and **f**, respectively. For abbreviations, see list

**Fig. 9:**
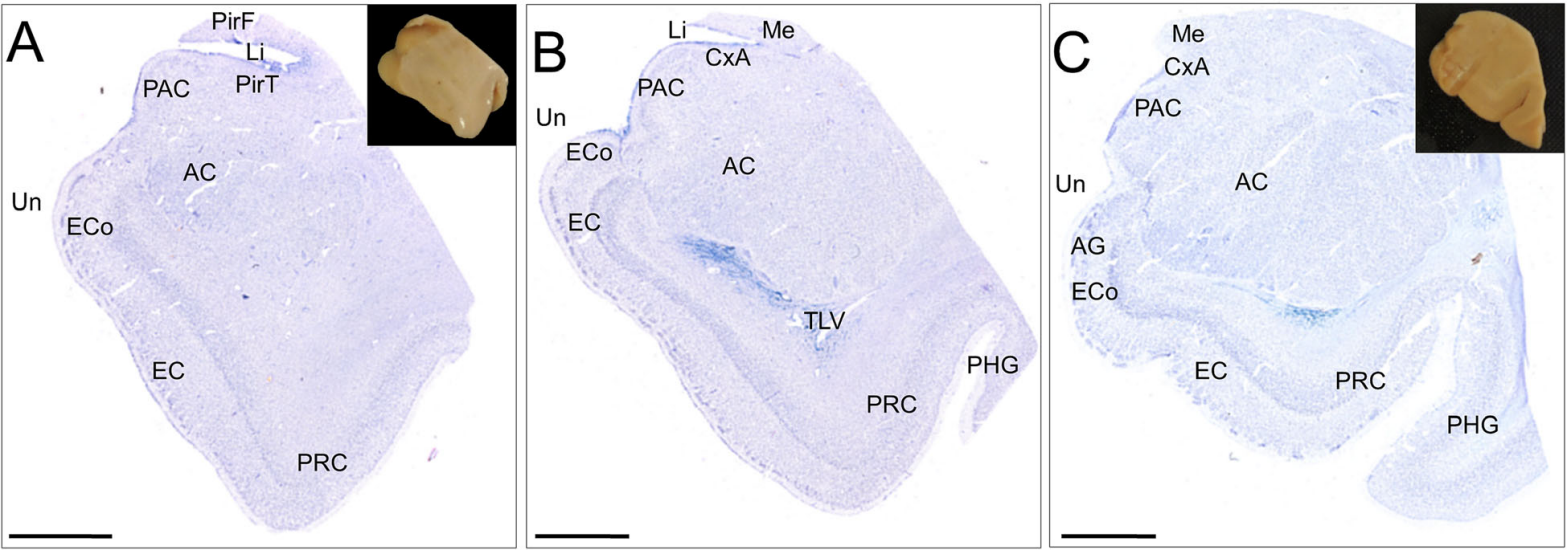
Nissl-stained coronal mosaic-reconstructed sections of the human temporal lobe illustrating olfactory structures (**a**, **b**, **c**). Panels **a** and **c** include pictures of tissue blocks from where sections were obtained. Calibration bar: 5000 μm for **a–c**. For abbreviations, see list

The intracranial components of the olfactory system can be macroscopically identified in magnetic resonance images (Figs. 3, 4 and 5) as well as macro- and microscopically in images of brain tissue blocks and mosaic-reconstructed Nissl-stained sections and 3D reconstructions (Figs. 6, 7, 8 and 9).

#### The olfactory epithelium

The olfactory epithelium is located at the dorsal and posterior portions of the nasal cavity [17] and can be distinguished from the respiratory epithelium by the presence of cilia (Fig. 2) [18]. In this epithelium, bipolar receptor cells, microvillar cells, sustentacular cells and basal cells can be distinguished [18], and receptor cells suffer a turnover process during adulthood [19]. These bipolar receptor cells display cilia to the nasal cavity and send axons (the fila olfactoria) that cross through the foramina of the cribriform plate of the ethmoid bone to reach the glomeruli of the olfactory bulb [14, 17, 18]. These receptor cells display pathological aggregates in patients with Alzheimer’s and Parkinson’s [20–23].

#### The olfactory bulb

The olfactory bulb constitutes the first cranial nerve but has some atypical characteristics [24, 25]. The axons from the olfactory receptors do not form a unique bundle; rather, after passing the ethmoid bone, the different *fila olfactoria* constitute the olfactory nerve. These axons are unmyelinated and covered by ensheathing glia. The bulb has a clear laminar structure (Fig. 6a) that includes the olfactory nerve layer, glomerular layer, external plexiform layer, mitral cell layer, internal plexiform layer, granule cell layer and stratum album (Fig. 6b) [12, 17, 26–28]. Axons from olfactory receptor cells establish synapses in spheres of neuropil in the bulb called glomeruli (asterisks in Fig. 6b). These axons are continuously replaced, and therefore, the circuitry of the olfactory bulb is remodeled. The olfactory nerve constitutes a potential and direct pathway for the entrance of viruses, neurotoxins and other xenobiotics, as well as for therapeutic agents targeting the brain. In the glomeruli, axons from olfactory receptor neurons establish synapses with apical dendrites of mitral and tufted cells. These projection cells are locally modulated by periglomerular and granule cells [29]. From the olfactory bulb (Figs. 3a, arrow in 3b, 6a), mitral and tufted cells send their axons to form a long bundle, the olfactory peduncle (arrowhead in Figs. 3b, 6a, c), coursing between the straight gyrus and the medial orbital gyrus (Figs. 3c, 4a, b, 7a), to join the basal frontal lobe and reach different olfactory structures: anterior olfactory nucleus, piriform cortex, olfactory tubercle, cortical amygdala, medial amygdala, cortex–amygdala transition zone and olfactory portion of the entorhinal cortex [12–14, 30] (Figs. 4, 5, 7, 8, 9).

#### The anterior olfactory nucleus

The human anterior olfactory nucleus is composed of at least seven divisions along the olfactory system (Fig. 2): bulbar (including several components) (Figs. 6a, d, 8a, b); intrapeduncular (Figs. 6c, e); retrobulbar (Figs. 7b, c, 8c–f); and anterior (Figs. 7d, e, 8c–f) and posterior (Figs. 7f, g, 8c–f) cortical portions with their medial and lateral components [12, 26, 31]. This organization into divisions appears to be exclusive to primates [12, 32] and humans [12, 26, 33], with all divisions being preferentially affected by proteinopathies [4, 31, 34–37].

#### The olfactory cortex

Along the olfactory system (Fig. 2), when the olfactory peduncle approaches the anterior perforated substance (Figs. 4a, b, 7a), it contacts the basal frontal lobe (Fig. 4c, d, 7b, c) and flattens out as the olfactory trigone, thus constituting the medial and lateral olfactory striae (Figs. 7d, e) [17]. The medial olfactory stria, much reduced, reaches the cortical anterior medial anterior olfactory nucleus and extends towards the diagonal band of Broca. The lateral olfactory stria reaches the cortical anterior lateral anterior olfactory nucleus, extending further laterally (Figs. 4e, f, 7d, e). Caudally, the olfactory tubercle, the cortical posterior medial anterior olfactory nucleus and its lateral divisions, and the frontal piriform cortex are reached (Figs. 7f, g, h). At the level of the limen insulae and beyond, in addition to some of the previous structures, the temporal piriform cortex, the olfactory entorhinal cortex, the periamygdaloid cortex, the cortex–amygdala transition zone and the medial amygdala (Figs. 5a–f, 9a–c) are also included among the olfactory-recipient structures. It should be noted that there is no direct evidence in humans of the exact extension of the olfactory cortex, but it has been studied through functional magnetic resonance imaging, diffusion tensor imaging and tractography and comparative neuroanatomical studies [12, 30, 32, 38–41].

### Alzheimer’s and Parkinson’s diseases

Although Alzheimer’s disease is mostly idiopathic (> 95% of patients suffer the sporadic form) [1], there is growing evidence of a genetic predisposition (60–80% attributable risk) [42]. Mutations in apolipoprotein E4 (APOE4) are the main risk factor, with the lifetime risk for Alzheimer’s disease being more than 50% for APOE4 homozygotes and 20–30% for APOE3 and APOE4 heterozygotes [43]. The disease has an estimated prevalence of 10–30% in the population > 65 years of age, with an incidence of 1–3% [1, 44, 45]. Clinical diagnosis is based on cognitive deficits [44], particularly anterograde (episodic) amnesia [46]. Pathophysiologically, it is a consequence of the imbalance in the production and clearance of the amyloid-β (Aβ) peptide from the extracellular space of the brain, which subsequently may induce (or be permissive of) tau aggregation by as yet unknown mechanisms [1, 45, 47, 48].

**Fig. 10:**
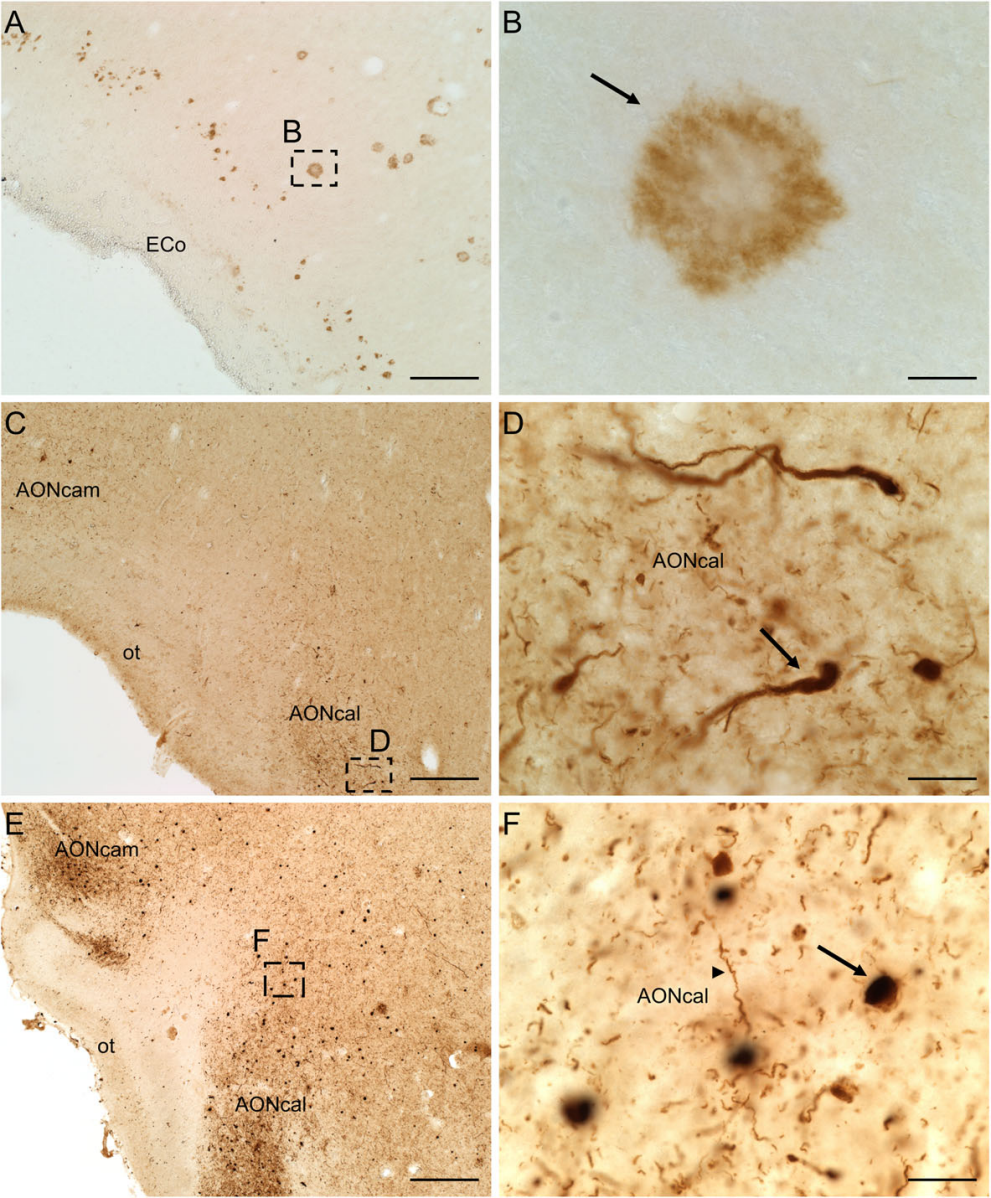
Immuno-stained sections of the human brain illustrating the labelling against amyloid-β peptide (**a**), tau protein (**c**) and α-synuclein protein (**e**). High-power images shown in **b** (arrow points to a plaque), **d** (arrow points to a dystrophic neurite) and **f** (arrow points to a Lewy body and arrowhead to a Lewy neurite) correspond to frames in **a**, **c** and **e**, respectively. Calibration bars: 250 μm for **a, c, e**, 20 μm for **b, d, f**. For abbreviations, see list

Neuritic plaques mostly composed of amyloid-β peptide (Fig. 10a, b) and neurofibrillary tangles of tau protein (10C, D) are the neuropathological hallmarks of the disease [45, 47–49]. These aggregates characterize a six-stage predictable sequence beginning in the locus coeruleus, olfactory bulb and (trans)entorhinal cortex and subsequently extending to the rest of the temporal cortex and other isocortical areas (Fig. 1) [45, 47, 49].

In Parkinson’s disease, two centuries after its nosologic description, research criteria for the diagnosis are still subject of debate [50]. Etiologically, most cases are idiopathic, with inherited genetic forms only accounting for a small percentage (5–10%) of diagnosed patients [2], particularly in monogenic forms of Parkinson’s disease. SNCA, which encodes the protein α-synuclein, was the first gene identified as being linked to the disease [51]. Mutations in LRRK and parkin are the most common causes of dominantly and recessively inherited cases, respectively [52]. Worldwide incidence estimates of Parkinson’s disease range from 5 to > 35 new cases per 100,000 individuals annually, with the global prevalence being 0.3% and increasing to > 3% in people over 80 years of age [2]. Clinical diagnosis is based on bradykinesia plus muscular rigidity and/or resting tremor and/or postural instability [52]. Nonmotor symptoms are gaining interest for early diagnosis [2]. Neuropathologically, Parkinson’s disease includes neuronal loss in the ventrolateral tier of the substantia nigra pars compacta and the corresponding striatal dopamine denervation and widespread intracellular α-synuclein accumulation [2, 53, 54].

Lewy bodies and neurites (Fig. 10e, f) [55] contain ubiquitin but are mostly made up of aggregated α-synuclein [56], which is encoded in a gene that has been identified in familial Parkinson’s disease [51]. These aggregates occur initially in cholinergic and monoaminergic brainstem neurons and in neurons in the olfactory system, and they are also found in the limbic and isocortical regions with disease progression (Fig. 1) [57, 58]. In comorbid patients also suffering from Alzheimer’s disease, α-synucleinopathy exhibits a different pattern concentrated mostly in limbic brain regions [59].

### Common traits of Alzheimer’s and Parkinson’s diseases

As described above, Alzheimer’s and Parkinson’s diseases have a number of etiology, symptomatology and treatment differences. Both disorders share, however, a long prodromal period during which most patients suffer hyposmia and associated proteinopathies that give rise to aggregates that accumulate initially and preferentially in olfactory structures.

#### Prodromal period: hyposmia

There is growing evidence that both Alzheimer’s [60–62] and Parkinson’s [63–67] have a long period of progression of years, even decades, before clinical diagnosis can be established. This therapeutic window should be exploited to improve early diagnosis.

Olfactory dysfunction has been proposed as an early marker of both Alzheimer’s [68] and Parkinson’s [69] diseases. This topic has recently gained some attention, but its use in clinical diagnosis is still not routine, due in part to the difficulty in distinguishing hyposmia (threshold and discriminative olfactory deficits) caused by different neurodegenerative diseases and aging [3, 70]. The neural substrates underlying hyposmia are largely unknown. It has been proposed damage in the olfactory epithelium, olfactory bulb and/or olfactory cortex or even involvement of centrifugal neuromodulator systems, such as the cholinergic system [3].

For Alzheimer’s disease, a prospective study including a cohort of 757 participants with a follow-up at 2 years and 4 years, using the University of Pennsylvania Smell Identification Test (UPSIT), suggested that odor identification was superior to verbal episodic memory deficits in predicting cognitive decline [71]. In the same vein, odor identification has been demonstrated to be useful for identifying Alzheimer’s disease pathology in healthy high-risk individuals [72]. A report among individuals with normal cognition, amnestic mild cognitive impairment, nonamnestic mild cognitive impairment and dementia indicates that olfactory impairment predicts amnestic mild cognitive impairment and progression to Alzheimer’s disease [73]. Analysis of odor identification, cognition and markers in cerebrospinal fluid revealed that lower odor identification scores correlated with increased tau concentrations. Odor identification impairment may therefore be used as a marker of neuronal damage rather than amyloid pathology [74]. It has been demonstrated that olfactory dysfunction in the presence of one or more APOE-ϵ4 alleles is associated with a high risk of cognitive decline [75, 76]. These data are supported by experimental models [77]. A recent review of olfactory and other sensory impairments as biomarkers concluded that odor identification, odor familiarity and odor recognition clearly allow discrimination between patients with Alzheimer’s disease, patients with mild cognitive impairment, those at risk of Alzheimer’s disease (amyloid-β deposition or genetic predisposition) and cognitively normal individuals [78]. Interestingly, a comparison between patients with Alzheimer’s disease-related cognitive impairment and Lewy body-related cognitive impairment revealed that cortical atrophy in the former and white matter abnormalities in the latter play key roles in olfactory deficits [79].

For Parkinson’s disease, olfactory dysfunction has been shown to be present in approximately 90% of preclinical cases and can precede the onset of motor symptoms by decades [80]. Prospective studies have also shown that hyposmia is correlated with at least a 10% increased risk of developing Parkinson’s disease [81], that it can predate clinical Parkinson’s disease by at least 4 years [82] and that, among diagnosed patients, severe olfactory dysfunction is a prodromal symptom of dementia associated with Parkinson’s disease [83]. Recent advances optimizing olfactory testing may help to generalize its use in the clinical evaluation of Parkinson’s disease [84].

#### Etiology: idiopathic diseases with early aggregates in the olfactory system

The etiologies of both diseases, although largely unknown, are substantially different, and although both are idiopathic, the contributions of different factors are still not fully understood. They share mitochondrial dysfunction, oxidative stress imbalance, perturbation of calcium homeostasis, neurotransmission alteration and protein misfolding that yields aggregates [1, 2].

In Alzheimer’s disease, tau accumulation is probably the best histopathological indicator of clinical progression [85]. Interestingly, early descriptions showed that the cortex of “associative” areas including the frontal, temporal and parietal lobes was severely involved, whereas motor, somatosensory and primary visual areas were virtually unaffected – with the exception of the olfactory system, which was invariably and massively affected [86, 87]. Following this observation, Braak’s proposed staging included neurofibrillary tangles and neuropil threads in the transentorhinal cortex (stages I–II), limbic structures (stages III–IV) and isocortex (stages V–VI) [88], with primary cortices being largely preserved [89]. The primary visual cortex, for instance, is not involved until stage VI, which is a diagnostic criterion. Improvement in this staging system using paraffin sections and hyperphosphorylated tau protein antibody allowed observation of the development of the earliest lesions in subcortical brain regions in the locus coeruleus [49]. Given their invariable and early involvement, the locus coeruleus and other structures within the olfactory system have been considered “hubs” for proteinopathy spreading (Fig. 1) [90].

Early reports on the olfactory bulbs in Alzheimer’s disease indicated the presence of neurofibrillary tangles, limited to the anterior olfactory nucleus, accompanied by cell loss [91]. Neuritic plaques were also found in the anterior olfactory nucleus, as well as neurofibrillary tangles and neuropil threads in the anterior olfactory nucleus and in other layers of the olfactory bulb [36]. Since then, a number of studies have confirmed these findings, suggesting that this pathology starts very early and correlates with Braak staging [92–98]. Amyloid-β and tau were also reported in the cortical anterior olfactory nucleus [34] and the piriform cortex [99]. Finally, tau and amyloid-β aggregates have also been described in dystrophic neurites of the olfactory epithelium of patients with Alzheimer’s disease, which is symptomatic of brain pathology [20–22]. As a result, the olfactory system has gained renewed interest in research into proteinopathies associated with Alzheimer’s disease [4, 100].

In Parkinson’s disease, very early immunoreactive Lewy bodies and neurites have been described in the dorsal glossopharyngeus–vagus complex and in the olfactory bulb, olfactory tract and anterior olfactory nucleus [101]. This initial description was followed by a complete staging proposal: 1) medulla oblongata (dorsal IX/X motor nucleus) and anterior olfactory nucleus; 2) medulla oblongata and pontine tegmentum (raphe nuclei and coeruleus–subcoeruleus complex); 3) midbrain (substantia nigra, pars compacta); 4) basal prosencephalon and mesocortex (transentorhinal region) and allocortex (CA2); 5) high-order association areas of the isocortex; and 6) first-order sensory association areas and premotor isocortex (Fig. 1) [57]. Less than 10% of cases examined (which, interestingly, had concomitant Alzheimer’s disease) showed a different pattern: olfactory structures and the amygdala were predominantly involved with a virtual absence of brainstem pathology [59, 102].

The pathology has historically been found to preferentially affect the olfactory system. Early reports described, using ubiquitin antibodies, Lewy bodies in the olfactory bulb and tract, particularly in the anterior olfactory nucleus [103]. Misrouted olfactory fibers in the external plexiform layer of the olfactory bulb formed glomerulus-like structures in Parkinson’s disease cases [104]. Immunohistochemistry against α-synuclein in patients with dementia, including those suffering from Parkinson’s disease, revealed Lewy-type pathology along the olfactory system, including not only the olfactory bulb and olfactory tract but also the anterior olfactory nucleus (cortical) and olfactory cortex [105]. The pathology has been suggested to be particularly abundant in the seven described divisions of the anterior olfactory nucleus [31, 35]. Among the primary olfactory cortex, the pathology was significantly more severe in the temporal division of the piriform cortex than in the frontal division of the piriform cortex, olfactory tubercle or anterior portions of the entorhinal cortex [106]. Reports on the olfactory bulb of aged people with different neuropathological diagnoses have revealed a high incidence of Lewy-body-related α-synucleinopathy that presumably extends from the periphery to the anterior olfactory nucleus [37]. In fact, olfactory bulb α-synucleinopathy is considered highly specific and highly sensitive for Lewy body disorders, to the point that it has been suggested that olfactory bulb biopsies be performed to confirm the diagnosis in subjects prior to surgical therapy [107]; however, there is controversy over this suggestion given the invasiveness of the procedure [108, 109]. Lewy body pathology has also been detected in the olfactory epithelium of patients with Parkinson’s disease [23], as has been the case for prion protein in Creutzfeldt-Jakob disease [110]. Therefore, the olfactory system is being regarded as of particular interest in the study of α-synucleinopathy in Parkinson’s disease [4, 30, 111].

### Morphometry: magnetic resonance imaging and stereological analysis

In Alzheimer’s disease, hippocampal and parahippocampal atrophy measured using magnetic resonance imaging have been widely reported as having a high correlation with cognitive and sensory tests [112]. The medial temporal lobe cortex, in particular the entorhinal cortex, is quite reduced, and this is correlated with episodic memory impairment [113–115]. Olfactory cortex degeneration has been associated with a decline in olfactory activity in Alzheimer’s disease and subjects with mild cognitive impairment [116]. Similarly, olfactory bulb atrophy has also been reported [117] and has been linked to atrophy of the medial temporal lobe [118]; however, this atrophy does not appear to be associated with olfactory dysfunction [119].

These findings have been paralleled by studies in postmortem tissue using stereological methods [120]. The CA1 hippocampal field (68%) [121] and layers IV and II of the entorhinal cortex (40–70% and 60–90%, respectively, depending on the severity of cases) [122] have been reported as the most distinctive regions regarding neuron loss. In fact, neuronal loss is detectable in mild Alzheimer’s disease but not in preclinical cases or those associated with normal aging [123, 124]. Volume and neuron numbers significantly decline with disease duration, which suggests that hippocampal atrophy is a result of neuronal loss [125]. For the oldest cases, the Clinical Dementia Rating appears to be more dependent on damage to other hippocampal subdivisions than on severe neurofibrillary tangle formation in the entorhinal cortex and CA1 field [126]. Other studies exploring the relationship between the Clinical Dementia Rating and hippocampal neuronal pathology have indicated a dissociation between the progression of neurofibrillary tangles and neuronal loss in the entorhinal cortex and CA1 field, and only a limited amount of cognitive dysfunction has been attributed to Alzheimer’s neuronal pathology in these areas [127, 128]. In the olfactory bulb, no changes in the total number of cells or the total number of mitral cells were reported, but rather a significant decrease in the volume of the olfactory bulb and in the total number of cells in the anterior olfactory nucleus [93]. Other studies have confirmed a significant volume decrease and an increase in periglomerular dopaminergic cells in Alzheimer’s patients compared to controls [129].

In Parkinson disease, voxel-based morphometry studies have demonstrated that olfactory dysfunction in Parkinson’s disease is related to olfactory-specific regions, namely, in the right amygdala and piriform cortex [130]. Olfactory dysfunction has been correlated not only with piriform but also with orbitofrontal cortex atrophy [131]. Other studies have explored the controversial association between olfactory performance and gray matter reduction in olfactory areas [132, 133]. A reduced disgust response has also been linked to atrophy of the piriform and orbitofrontal cortex [134].

At the same time, morphometric studies in postmortem human tissue have revealed cell loss of dopaminergic neurons in the ventral tier of the substantia nigra pars compacta due to aging [135, 136] and dementia with Lewy bodies [137], and this is particularly marked in Parkinson’s disease [138, 139]. This cell loss appears to be progressive during the prodromal period, but the correlation with Lewy body pathology is unclear [140, 141]. Global counting of neocortical neurons does not reveal significant cell loss in Parkinson’s disease compared with controls [142]. In limbic structures, however, the situation is contradictory. In the amygdala, the volume was reduced by 20%, and cell loss (in parallel with increased Lewy pathology) was significant in the cortical and basolateral nuclei; clinically, this was correlated with anosmia and visual hallucinations, respectively [143]. In contrast, in the different hippocampal fields [144], no differences have been found in the numbers of neurons or glial cells [145]. Interestingly, in olfactory structures, the data are more uniform. Profound cell loss has been reported in the olfactory bulb and tract, particularly in the anterior olfactory nucleus, showing a strong correlation with disease duration and paralleling Lewy pathology [146]. Data on cell losses in the olfactory bulb and its correlation with Lewy pathology have been corroborated [140]. No significant volume changes have been reported, but an increase in dopaminergic cells has been cited as a compensatory mechanism [129, 147], which is significantly higher in males [148].

### Proteinopathies

Transcriptomic [149] and proteomic [150] data within the Human Brain Proteome Project are very useful for understanding proteinopathies in the olfactory system. This kind of analysis has been particularly useful in neurodegenerative diseases [151], including Alzheimer’s and Parkinson’s diseases [152]. In the olfactory bulb of the APP/PS1 model of Alzheimer’s disease, early cytoskeletal disruption and synaptic impairment have been reported, whereas studies in the Tg2576 model have allowed us to further characterize the dysregulation of molecular homeostasis [153, 154]. Analysis of human samples has allowed us to characterize proteomic changes along staging in Alzheimer’s [155–157] and Parkinson’s [158, 159] diseases.

Neuritic plaques mainly, but not solely, consist of amyloid-β aggregates. Amyloid-β is derived through the proteolytic cleavage of amyloid precursor protein (APP) by a heterogeneous family of enzymes (γ-secretases and β-secretases, including presenilin 1 and 2), giving rise to peptides ranging from 38 to 43 amino acids. Several lines of evidence have suggested that amyloid-β accumulation and conformational changes from an α-helix to a β-sheet structure may be crucial in the pathogenesis of the disease [1].

Tau is a protein highly present in neurons and originally described by its ability to bind and stabilize microtubules. Beyond that, tau mediates axonal transport, synaptic function and signaling pathways. Knowledge of its physiological roles and posttranslational modifications is necessary to understand its implications in the pathogenesis of Alzheimer’s disease [160].

The normal function of α-synuclein 140-amino-acid protein is not well understood, but it is present in the cytosol, likely the mitochondria and the nucleus, and it probably participates in synaptic-vesicle fusion, trafficking inside the cell and mitochondrial function. During the pathogenic process, α-synuclein misfolds to form β-rich sheets, acquires toxic traits and becomes insoluble when monomers form oligomers, protofibrils and eventually fibrils that yield Lewy pathology [2, 161].

### Prion-like spreading

Over time, the prion-like hypothesis in neurodegenerative diseases has gained interest, as it is supported by data obtained in postmortem tissue, patients and experimental models in vitro and in vivo. In Alzheimer’s disease, there is growing evidence for both amyloid-β and tau acting in a prion-like manner [162], as well as for α-synuclein in Parkinson’s disease [163–171]. In Parkinson’s disease in particular, the debate [172] for [173] and against – considering that this disease cannot be explained simply from a prionoid perspective – [174] is ongoing.

There was a paradigmatic change in the approach to neurodegenerative diseases after the proposal of neuropathological staging in Alzheimer’s [49, 88] and Parkinson’s [57, 175] diseases. This proposal was, in most cases, reproducible, accumulative and predictable, and it followed a sequence of neuronal connected regions that perfectly matched the idea of prion-like proteinopathy spreading [176]. For Alzheimer’s disease, the idea of a possible transmission of tau from the locus coeruleus to the transentorhinal cortex via neuron-to-neuron transmission and transsynaptic transport has been proposed after the observation of pretangles within noradrenergic coeruleus projection neurons in the absence of any pathology in the medial temporal lobe [176, 177]. For Parkinson’s disease, the proposed route of transmission would begin in the gastrointestinal mucosa and, via postganglionic enteric neurons, would enter the central nervous system retrogradely through poorly myelinated vagal neurons [176, 178, 179]. Alternatively, a nasal route through the olfactory epithelium and bulb – the olfactory vector hypothesis [180] – was also proposed, constituting the dual-hit hypothesis according to which spread may occur anterogradely through the olfactory epithelium and/or retrogradely through the intestinal mucosa [181, 182]. This latter possibility has been reinforced by the fact that truncal vagotomy appears to be protective against this disorder [183], although human autopsy evidence does not support this possibility and the debate remains open [184].

In vitro and in vivo data have shown that wild-type human tau protein, but not mutated tau, injected in the hippocampus of rats is able to propagate to olfactory structures through transsynaptic mechanisms [185]. In vitro experiments with α-synuclein support the notion that protein aggregation is not the primary cause of cytotoxicity [186]. Lewy body extracts from patients have demonstrated their toxicity and spreading capacity among neurons and astrocytes [187]. Injections in the olfactory bulb [188] or anterior olfactory nucleus [16] induced distant or contralateral α-synucleinopathy, respectively.

### Connectome and pathology

**Fig. 11:**
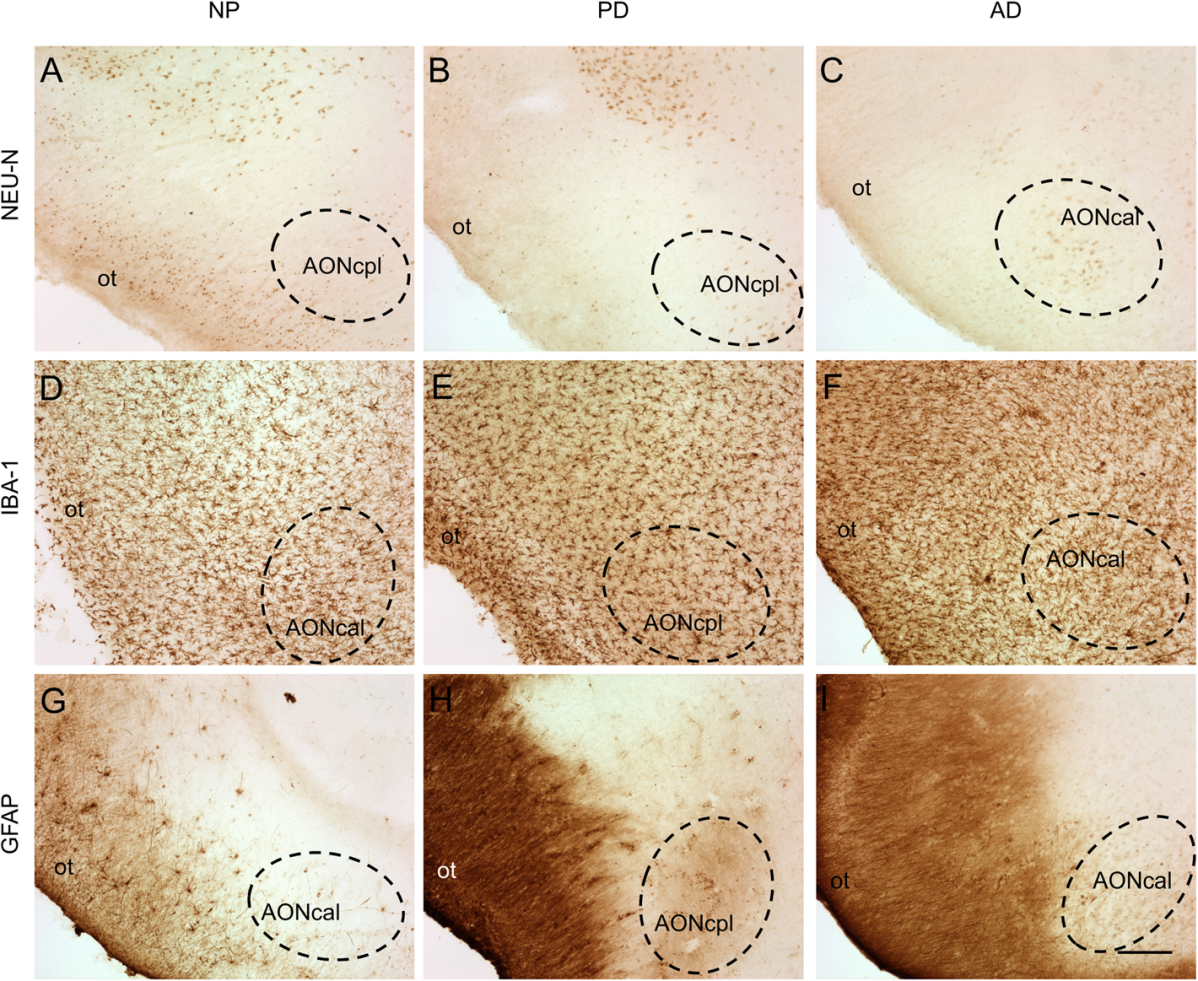
Immuno-stained sections of the human brain from non-pathological (**a**, **d**, **g**), Parkinson’s disease (B, E, H) and Alzheimer’s disease (**c**, **f**, **i**) cases illustrating the labelling against markers of neurons (Neu-N) (**a**, **b**, **c**), microglia (Iba-1) (**d**, **e**, **f**) and astroglia (GFAP) (**g**, **h**, **i**). Dashed lines indicate the boundaries of different portions of the anterior olfactory nucleus. Calibration bar: 125 μm for **a**–**i**. For abbreviations, see list

Neurodegenerative diseases can only be approached from a global perspective that encompasses the micro- to the macroscale. Neuronal activity is affected at many levels, including genetic, molecular, synaptic, cellular (neurons and glia), local circuits and networks [189]. In the previous sections, microscale levels have been considered. In this section, cellular and connectomic levels will be discussed, focusing on the selective involvement (vulnerability or resistance) of certain neuronal (Figs. 11a–c) and glial (Figs. 11d–i) populations and how nodes and hubs within the olfactory system through their connections can help explain the pathophysiology of neurodegenerative diseases (Fig. 1).

**Fig. 12:**
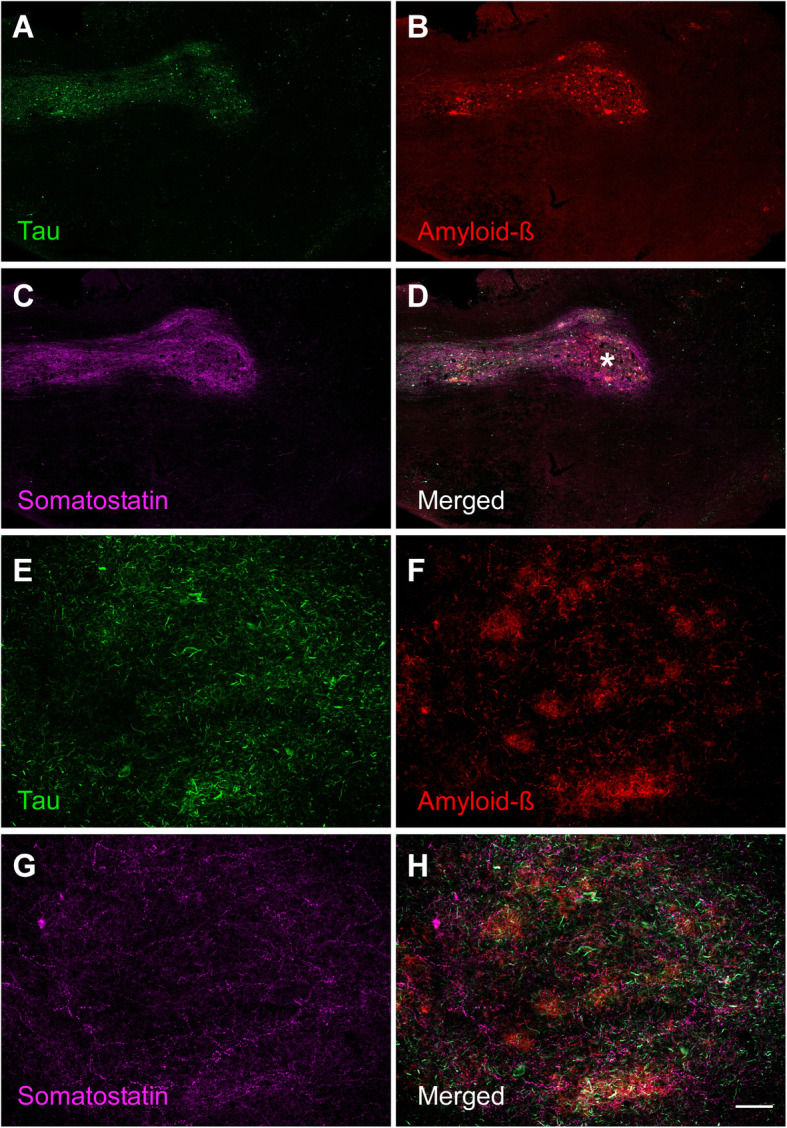
Immuno-fluorescent sections of the human olfactory bulb from an Alzheimer’s disease case illustrating the labelling against markers of tau protein (**a**), amyloid-β (**b**) and somatostatin (**c**) as well as the merged image (**d**). Calibration bar: 500 μm for **a**–**d**, 60 μm for **e**-**h**. For abbreviations, see list

In Alzheimer’s and Parkinson’s diseases, the specific cell types that are prone to developing proteinaceous inclusions are projection cells with long, unmyelinated or sparsely myelinated axons. Among the olfactory cortical areas, in Alzheimer’s stages I–II and in Parkinson’s stage 4, the pathological process reaches the entorhinal cortex and hippocampus, progressing thereafter to the isocortex. Regarding neuronal vulnerability, data in the literature are not homogeneous in the olfactory system [100]. Some data conclude the resistance of interneuron populations expressing calcium-binding proteins in Alzheimer’s disease in the pirirform cortex [99], whereas others suggest a selective vulnerability depending on entorhinal subfields [190]. Several investigations have pointed out the high vulnerability of d somatostatinergic neurons in the olfactory system in Alzheimer’s disease [34, 99, 191] (Figs. 12 A–D). Glial cells have always been regarded as subordinates of neuronal function, either with immune and phagocytic capacity (microglia) (Figs. 11d–f) or the responsible of homeostasis and metabolic neuronal maintenance, including establishment of the blood–brain barrier (astroglia) (Figs. 11g–i). The role of glial cells on connectomic interactions is just beginning to be envisaged.

The term connectome, first used more than one decade ago [192], aims to describe the structural and functional connectivity of the human brain among connected (nodes) and highly connected (hubs) regions from an -omic perspective and to explore similarities and differences across disorders, including disconnectivity [7]. The connectomic approach has allowed mapping lesion symptoms into brain networks [193] as well as identifying hubs, such as the amygdala [194] and the medial temporal lobe [195], namely, the hippocampus [196], which are generally implicated in the anatomy of brain disorders such as depression and Alzheimer’s disease, respectively.

Imaging techniques for visualizing the pathophysiology of Alzheimer’s disease in patients have revealed that the earliest deposits of amyloid-β appear in the medial parietal cortex in the first stages of the disease, whereas aggregates of tau occur earlier in the medial temporal lobe in cognitively healthy older people. It is debated whether the first deposits of tau in the medial temporal lobe represent the early stages of the disease or are somewhat innocuous until the presence of amyloid-β [197]. Similarly, it has been proposed that amyloid-β oligomers initially produced in isocortical neurons, including the synaptic terminal on entorhinal cortex neurons, may subsequently catalyze the formation of tau oligomers that could spread to the hippocampal formation, locus coeruleus, basal forebrain cholinergic neurons, raphe nuclei, and back to the isocortex [198]. Alternatively, it has been proposed that one potential trigger for tau protein hyperphosphorylation and conformation change may be a nonendogenous pathogen that develops tau lesions, beginning “in phylogenetically late-appearing and ontogenetically late-maturing neurons that are connected via their axons” [90] (Fig. 1).

Neuropathological analyses have illustrated that tau aggregates form early and preferentially occur in the locus coeruleus, olfactory bulb/anterior olfactory nucleus, amygdala [199] and entorhinal/transentorhinal cortex – three potential hubs. The latter constitutes a bottleneck for information entrance to and retrieval from the cortex through the hippocampus that likely helps to explain its relevance in the disease [90]. On the other hand, the olfactory bulb and, particularly, the different divisions of the anterior olfactory nucleus, are also early locations that are preferentially affected by Alzheimer’s pathology, likely due to their reciprocal and contralateral connections with olfactory structures as well as more distant regions such as the temporal cortex [16, 32, 100]. It is important to note that there is a part of the entorhinal cortex that receives direct olfactory information [30, 38], thus constituting a direct connection among hubs in the olfactory system and those in the entorhinal–hippocampal–cortical loop, thus allowing reciprocal interactions between the earliest involved regions in Alzheimer’s disease (Fig. 1).

In Parkinson’s disease, postmortem investigations have allowed a detailed reconstruction of the potential neuronal connections involved, including the central [55, 57] and peripheral nervous systems [200], in progressive stages: 1) enteric nervous system, olfactory bulb and motor nucleus of the vagus/glossopharyngeal nerves; 2) raphe nuclei and locus coeruleus; 3) substantia nigra and amygdala; 4) hippocampus and temporal mesocortex; 5) high-order association cortex; and 6) primary sensory cortex. Accordingly, structures in the first stages constitute potential hubs for α-synuclein spreading [30]. In the amygdaloid complex, for instance, the central nucleus appears to be crucial for synucleinopathy propagation in the forebrain [55, 199, 201]. Given their position and connectivity, the olfactory bulb [202] and particularly the anterior olfactory nucleus [30] are crucial for centripetal spreading of Lewy pathology (Fig. 1).

In fact, potential digestive mucosa pathology can retrogradely spread to the dorsal motor nucleus of the vagus nerve. Within the amygdala, the central nucleus is the output component of the amygdaloid complex, intervening in sympathetic and parasympathetic responses mediated by the amygdala. Therefore, the central amygdaloid nucleus is connected to the dorsal motor nucleus of the vagus. Interestingly, the central nucleus shows denser Lewy pathology among the amygdaloid nuclei [143, 201]. On the other hand, the amygdala (cortical) receives direct olfactory inputs, and the different amygdaloid nuclei are highly interconnected, including the periamygdaloid cortex and the central nucleus. Thus, another important interconnection occurs between hubs in the amygdala–parasympathetic system and those of the olfactory system. This second network, of course, is also primarily connected to the entorhinal–hippocampal–cortical cluster [30], which may help explain cases of Lewy pathology concentrated in the forebrain when Alzheimer copathology occurs (Fig. 1) [59, 102].

## Conclusions

Alzheimer’s and Parkinson’s diseases are prevalent neurodegenerative disorders with a long prodromal period during which preclinical manifestations such as hyposmia occur. Associated proteinopathies are amyloid-β and tau and α-synuclein. Aggregates occur in the olfactory system, particularly in the anterior olfactory nucleus, early and in a preferential manner. The olfactory system is composed of a number of mostly cortical structures along the frontal and temporal lobes. Magnetic resonance imaging data and volumetric analyses have revealed some volume changes that must be complemented by histological reports to assess whether morphometric changes are due to neuronal and/or glial loss or parenchymal remodeling. Prion-like hypotheses applied to proteinopathies associated with Alzheimer’s and Parkinson’s diseases have suggested that misfolded proteins can “seed” and induce misfolding of native proteins, “spreading” through neurons and glia. Accumulated data from patients, postmortem tissue and in vivo and in vitro experiments support this hypothesis, whereas arguments against it highlight that direct proof is still missing. The connectomic perspective explores the factors providing resistance or vulnerability to neuronal populations and the positive and/or negative role of protein spreading. Hubs in the olfactory system (e.g., the anterior olfactory nucleus, amygdala and entorhinal cortex) are highly interconnected with hubs in the entorhinal–hippocampal–cortical and amygdala–parasympathetic clusters, which are essential for protein spreading in Alzheimer’s and Parkinson’s diseases, respectively. These hubs within the olfactory system should be particularly considered in future diagnostic, prognostic and therapeutic approaches using molecular imaging with PET.

## Data Availability

Not applicable.

## Abbreviations

AC: Amygdaloid complex
Acc: Nucleus accumbens
AG: Ambiens gyrus
AON: Anterior olfactory nucleus
AONb: Anterior olfactory nucleus, bulbar
AONc: Anterior olfactory nucleus, cortical
AONca: Anterior olfactory nucleus, cortical anterior
AONcal: Anterior olfactory nucleus, cortical anterior lateral
AONcam: Anterior olfactory nucleus, cortical anterior medial
AONcp: Anterior olfactory nucleus, cortical posterior
AONcpl: Anterior olfactory nucleus, cortical posterior lateral
AONcpm: Anterior olfactory nucleus, cortical posterior medial
AONi: Anterior olfactory nucleus, intrapeduncular
AONrb: Anterior olfactory nucleus, retrobulbar
CA1-CA3: Hippocampal fields
cc: Corpus callosum
CxA: Cortex–amygdala transition zone
db: Diagonal band (of Broca)
EC: Entorhinal cortex
ECo: Entorhinal cortex, olfactory
EPL: External plexiform layer
FL: Frontal lobe
FLAIR: Fluid Attenuated Inversion Recovery.
FLV: Frontal horn of lateral ventricle
GCL: Granule cell layer
GD: Dentate gyrus
GL: Glomerular layer
H: Hippocampus
IG: Insular gyrus
IPL: Internal plexiform layer
LC: Locus coeruleus
Li: Limen insulae
lolf: Lateral olfactory radiation
MCL: Mitral cell layer
Me: Medial amygdala
molf: Medial olfactory radiation
MOrG: Medial orbital gyrus
OB: Olfactory bulb
OE: Olfactory epithelium
ONL: Outer nerve layer
OP: Olfactory peduncle
opt: Optic tract
ot: Olfactory tract
OT: Olfactory tubercle
ox: Optic chiasm
PAC: Periamygdaloid cortex
PET: Positron emission tomography.
PHG: Parahippocampal gyrus
Pir: Piriform cortex
PirF: Piriform cortex, frontal
PirT: Piriform cortex, temporal
PMOL: Posteromedial orbital lobule
PRC: Perirhinal cortex
R: Raphe nuclei
S: Subicular complex
SA: Stratum album
SG: Straight gyrus
SN: Substantia nigra
TLV: Temporal horn of the lateral ventricle
TP: Temporal pole
Un: Uncus
X: Nucleus of the vagus nerve

## References

[1] Masters CL, Bateman R, Blennow K, Rowe CC, Sperling RA, Cummings JL (2015). Alzheimer's disease. Nat Rev Dis Primers.

[2] Poewe W, Seppi K, Tanner CM, Halliday GM, Brundin P, Volkmann J, Schrag AE, Lang AE (2017). Parkinson disease. Nat Rev Dis Primers.

[3] Doty RL (2017). Olfactory dysfunction in neurodegenerative diseases: is there a common pathological substrate?. Lancet Neurol.

[4] Attems J, Walker L, Jellinger KA (2014). Olfactory bulb involvement in neurodegenerative diseases. Acta Neuropathol.

[5] Jucker M, Walker LC (2013). Self-propagation of pathogenic protein aggregates in neurodegenerative diseases. Nature.

[6] Perry VH, Holmes C (2014). Microglial priming in neurodegenerative disease. Nat Rev Neurol.

[7] van den Heuvel MP, Sporns O (2019). A cross-disorder connectome landscape of brain dysconnectivity. Nat Rev Neurosci.

[8] Nieuwenhuys R, Voogd J, van Huijzen C (2008). The human central nervous system.

[9] Pena-Melian A (2019). Cabello-de la Rosa JP, Gallardo-Alcaniz MJ, Vaamonde-Gamo J, Relea-Calatayud F, Gonzalez-Lopez L, Villanueva-Anguita P, Flores-Cuadrado A, Saiz-Sanchez D, Martinez-Marcos A: Cranial Pair 0: The Nervus Terminalis. Anat Rec (Hoboken).

[10] Ubeda-Banon I, Pro-Sistiaga P, Mohedano-Moriano A, Saiz-Sanchez D, de la Rosa-Prieto C, Gutierrez-Castellanos N, Lanuza E, Martinez-Garcia F, Martinez-Marcos A (2011). Cladistic analysis of olfactory and vomeronasal systems. Front Neuroanat.

[11] Doty RL (2003). Handbook of olfaction and gustation.

[12] Price JL, Paxinos G (1990). Olfactory System. The Human Nervous System.

[13] van Hartevelt TJ, Kringelbach ML, Mai JK, Paxinos G (2012). The Olfactory System. The Human Nervous System.

[14] Martinez-Marcos A (2009). On the organization of olfactory and vomeronasal cortices. Prog Neurobiol.

[15] Mohedano-Moriano A, de la Rosa-Prieto C, Saiz-Sanchez D, Ubeda-Banon I, Pro-Sistiaga P, de Moya-Pinilla M, Martinez-Marcos A (2012). Centrifugal telencephalic afferent connections to the main and accessory olfactory bulbs. Front Neuroanat.

[16] Flores-Cuadrado A, Saiz-Sanchez D, Mohedano-Moriano A, Martinez-Marcos A, Ubeda-Banon I (2019). Neurodegeneration and contralateral alpha-synuclein induction after intracerebral alpha-synuclein injections in the anterior olfactory nucleus of a Parkinson's disease A53T mouse model. Acta Neuropathol Commun.

[17] Standring S (2008). Gray's anatomy. The anatomical basis for clinical practice. Fortieth edn.

[18] Doty RL (2012). Olfaction in Parkinson's disease and related disorders. Neurobiol Dis.

[19] Graziadei PP, Graziadei GA (1979). Neurogenesis and neuron regeneration in the olfactory system of mammals. I. Morphological aspects of differentiation and structural organization of the olfactory sensory neurons. J Neurocytol.

[20] Arnold SE, Lee EB, Moberg PJ, Stutzbach L, Kazi H, Han LY, Lee VM, Trojanowski JQ (2010). Olfactory epithelium amyloid-beta and paired helical filament-tau pathology in Alzheimer disease. Ann Neurol.

[21] Arnold SE, Smutzer GS, Trojanowski JQ, Moberg PJ (1998). Cellular and molecular neuropathology of the olfactory epithelium and central olfactory pathways in Alzheimer's disease and schizophrenia. Ann N Y Acad Sci.

[22] Lee JH, Goedert M, Hill WD, Lee VM, Trojanowski JQ (1993). Tau proteins are abnormally expressed in olfactory epithelium of Alzheimer patients and developmentally regulated in human fetal spinal cord. Exp Neurol.

[23] Saito Y, Shioya A, Sano T, Sumikura H, Murata M, Murayama S (2016). Lewy body pathology involves the olfactory cells in Parkinson's disease and related disorders. Mov Disord.

[24] Martinez-Marcos A, Sanudo JR (2019). Cranial nerves: morphology and clinical relevance. Anat Rec (Hoboken).

[25] Martinez-Marcos A, Sanudo JR (2019). Cranial nerves: phylogeny and ontogeny. Anat Rec (Hoboken).

[26] Crosby EC, Humphrey T (1941). Studies of the vertebrate telencephalon. II. The nuclear pattern of the anterior olfactory nucleus, tuberculum olfactorium and the amygdaloid complex in adult man. J Comp Neurol.

[27] Smith RL, Baker H, Kolstad K, Spencer DD, Greer CA (1991). Localization of tyrosine hydroxylase and olfactory marker protein immunoreactivities in the human and macaque olfactory bulb. Brain Res.

[28] Smith RL, Baker H, Greer CA (1993). Immunohistochemical analyses of the human olfactory bulb. J Comp Neurol.

[29] Crespo C, Liberia T, Blasco-Ibanez JM, Nacher J, Varea E (2019). Cranial pair I: the olfactory nerve. Anat Rec (Hoboken).

[30] Ubeda-Banon I, Saiz-Sanchez D, de la Rosa-Prieto C (2014). Martinez-Marcos a: alpha-Synuclein in the olfactory system in Parkinson's disease: role of neural connections on spreading pathology. Brain Struct Funct.

[31] Ubeda-Banon I, Saiz-Sanchez D, de la Rosa-Prieto C, Argandona-Palacios L, Garcia-Munozguren S (2010). Martinez-Marcos a: alpha-Synucleinopathy in the human olfactory system in Parkinson's disease: involvement of calcium-binding protein- and substance P-positive cells. Acta Neuropathol.

[32] Mohedano-Moriano A, Martinez-Marcos A, Munoz M, Arroyo-Jimenez MM, Marcos P, Artacho-Perula E, Blaizot X, Insausti R (2005). Reciprocal connections between olfactory structures and the cortex of the rostral superior temporal sulcus in the Macaca fascicularis monkey. Eur J Neurosci.

[33] Ohm TG, Muller H, Braak E (1991). Calbindin-D-28k-like immunoreactive structures in the olfactory bulb and anterior olfactory nucleus of the human adult: distribution and cell typology--partial complementarity with parvalbumin. Neuroscience.

[34] Saiz-Sanchez D, Ubeda-Banon I, de la Rosa-Prieto C, Argandona-Palacios L, Garcia-Munozguren S, Insausti R, Martinez-Marcos A (2010). Somatostatin, tau, and beta-amyloid within the anterior olfactory nucleus in Alzheimer disease. Exp Neurol.

[35] Ubeda-Banon I, Flores-Cuadrado A, Saiz-Sanchez D, Martinez-Marcos A (2017). Differential effects of Parkinson's disease on interneuron subtypes within the human anterior olfactory nucleus. Front Neuroanat.

[36] Ohm TG, Braak H (1987). Olfactory bulb changes in Alzheimer's disease. Acta Neuropathol.

[37] Sengoku R, Saito Y, Ikemura M, Hatsuta H, Sakiyama Y, Kanemaru K, Arai T, Sawabe M, Tanaka N, Mochizuki H (2008). Incidence and extent of Lewy body-related alpha-synucleinopathy in aging human olfactory bulb. J Neuropathol Exp Neurol.

[38] Insausti R, Marcos P, Arroyo-Jimenez MM, Blaizot X, Martinez-Marcos A (2002). Comparative aspects of the olfactory portion of the entorhinal cortex and its projection to the hippocampus in rodents, nonhuman primates, and the human brain. Brain Res Bull.

[39] Ibarretxe-Bilbao N, Junque C, Marti MJ, Valldeoriola F, Vendrell P, Bargallo N, Zarei M, Tolosa E (2010). Olfactory impairment in Parkinson's disease and white matter abnormalities in central olfactory areas: a voxel-based diffusion tensor imaging study. Mov Disord.

[40] Woodward MR, Dwyer MG, Bergsland N, Hagemeier J, Zivadinov R, Benedict RH, Szigeti K (2017). Olfactory identification deficit predicts white matter tract impairment in Alzheimer's disease. Psychiatry Res Neuroimaging.

[41] Zald DH, Pardo JV (2000). Functional neuroimaging of the olfactory system in humans. Int J Psychophysiol.

[42] Dourlen P, Kilinc D, Malmanche N, Chapuis J, Lambert JC (2019). The new genetic landscape of Alzheimer's disease: from amyloid cascade to genetically driven synaptic failure hypothesis? Acta Neuropathol.

[43] Genin E, Hannequin D, Wallon D, Sleegers K, Hiltunen M, Combarros O, Bullido MJ, Engelborghs S, De Deyn P, Berr C (2011). APOE and Alzheimer disease: a major gene with semi-dominant inheritance. Mol Psychiatry.

[44] Scheltens P, Blennow K, Breteler MM, de Strooper B, Frisoni GB, Salloway S, Van der Flier WM (2016). Alzheimer's disease. Lancet.

[45] Vinters HV (2015). Emerging concepts in Alzheimer's disease. Annu Rev Pathol.

[46] Tromp D, Dufour A, Lithfous S, Pebayle T, Despres O (2015). Episodic memory in normal aging and Alzheimer disease: insights from imaging and behavioral studies. Ageing Res Rev.

[47] Goedert M, Spillantini MG (2006). A century of Alzheimer's disease. Science.

[48] Thal DR, Walter J, Saido TC, Fandrich M (2015). Neuropathology and biochemistry of Abeta and its aggregates in Alzheimer's disease. Acta Neuropathol.

[49] Braak H, Alafuzoff I, Arzberger T, Kretzschmar H, Del Tredici K (2006). Staging of Alzheimer disease-associated neurofibrillary pathology using paraffin sections and immunocytochemistry. Acta Neuropathol.

[50] Postuma RB, Berg D, Stern M, Poewe W, Olanow CW, Oertel W, Obeso J, Marek K, Litvan I, Lang AE (2015). MDS clinical diagnostic criteria for Parkinson's disease. Mov Disord.

[51] Polymeropoulos MH, Lavedan C, Leroy E, Ide SE, Dehejia A, Dutra A, Pike B, Root H, Rubenstein J, Boyer R (1997). Mutation in the alpha-synuclein gene identified in families with Parkinson's disease. Science.

[52] Kalia LV, Lang AE (2015). Parkinson's disease. Lancet.

[53] Obeso JA, Stamelou M, Goetz CG, Poewe W, Lang AE, Weintraub D, Burn D, Halliday GM, Bezard E, Przedborski S (2017). Past, present, and future of Parkinson's disease: a special essay on the 200th anniversary of the shaking palsy. Mov Disord.

[54] Dickson DW, Braak H, Duda JE, Duyckaerts C, Gasser T, Halliday GM, Hardy J, Leverenz JB, Del Tredici K, Wszolek ZK, Litvan I (2009). Neuropathological assessment of Parkinson's disease: refining the diagnostic criteria. Lancet Neurol.

[55] Goedert M, Spillantini MG, Del Tredici K, Braak H (2013). 100 years of Lewy pathology. Nat Rev Neurol.

[56] Spillantini MG, Schmidt ML, Lee VM, Trojanowski JQ, Jakes R, Goedert M (1997). Alpha-synuclein in Lewy bodies. Nature.

[57] Braak H, Del Tredici K, Rub U, de Vos RA, Jansen Steur EN, Braak E (2003). Staging of brain pathology related to sporadic Parkinson's disease. Neurobiol Aging.

[58] Braak H, Bohl JR, Muller CM, Rub U, de Vos RA, Del Tredici K. Stanley Fahn Lecture 2005: The staging procedure for the inclusion body pathology associated with sporadic Parkinson's disease reconsidered. Mov Disord. 2006(21):2042–51. 10.1002/mds.21065 17078043

[59] Toledo JB, Gopal P, Raible K, Irwin DJ, Brettschneider J, Sedor S, Waits K, Boluda S, Grossman M, Van Deerlin VM (2016). Pathological alpha-synuclein distribution in subjects with coincident Alzheimer's and Lewy body pathology. Acta Neuropathol.

[60] Dubois B, Hampel H, Feldman HH, Scheltens P, Aisen P, Andrieu S, Bakardjian H, Benali H, Bertram L, Blennow K (2016). Preclinical Alzheimer's disease: definition, natural history, and diagnostic criteria. Alzheimers Dement.

[61] Sheng C, Huang Y, Han Y (2018). Dissection of prodromal Alzheimer's disease. Front Biosci (Landmark Ed).

[62] Sperling RA, Karlawish J, Johnson KA (2013). Preclinical Alzheimer disease-the challenges ahead. Nat Rev Neurol.

[63] Obeso JA, Rodriguez-Oroz MC, Goetz CG, Marin C, Kordower JH, Rodriguez M, Hirsch EC, Farrer M, Schapira AH, Halliday G (2010). Missing pieces in the Parkinson's disease puzzle. Nat Med.

[64] Hawkes CH (2008). The prodromal phase of sporadic Parkinson's disease: does it exist and if so how long is it?. Mov Disord.

[65] Postuma RB, Berg D (2019). Prodromal Parkinson's disease: the decade past, the decade to come. Mov Disord.

[66] Schapira AHV, Chaudhuri KR, Jenner P (2017). Non-motor features of Parkinson disease. Nat Rev Neurosci.

[67] Berg D, Postuma RB, Adler CH, Bloem BR, Chan P, Dubois B, Gasser T, Goetz CG, Halliday G, Joseph L (2015). MDS research criteria for prodromal Parkinson's disease. Mov Disord.

[68] Burns A (2000). Might olfactory dysfunction be a marker of early Alzheimer's disease?. Lancet.

[69] Doty RL, Deems DA, Stellar S (1988). Olfactory dysfunction in parkinsonism: a general deficit unrelated to neurologic signs, disease stage, or disease duration. Neurology.

[70] Benarroch EE (2010). Olfactory system: functional organization and involvement in neurodegenerative disease. Neurology.

[71] Devanand DP, Lee S, Manly J, Andrews H, Schupf N, Doty RL, Stern Y, Zahodne LB, Louis ED, Mayeux R (2015). Olfactory deficits predict cognitive decline and Alzheimer dementia in an urban community. Neurology.

[72] Lafaille-Magnan ME, Poirier J, Etienne P, Tremblay-Mercier J, Frenette J, Rosa-Neto P, Breitner JCS, Group P-AR (2017). Odor identification as a biomarker of preclinical AD in older adults at risk. Neurology.

[73] Roberts RO, Christianson TJ, Kremers WK, Mielke MM, Machulda MM, Vassilaki M, Alhurani RE, Geda YE, Knopman DS, Petersen RC (2016). Association between olfactory dysfunction and amnestic mild cognitive impairment and Alzheimer disease dementia. JAMA Neurol.

[74] Reijs BLR, Ramakers I, Elias-Sonnenschein L, Teunissen CE, Koel-Simmelink M, Tsolaki M, Wahlund LO, Waldemar G, Hausner L, Johannsen P (2017). Relation of odor identification with Alzheimer's disease markers in cerebrospinal fluid and cognition. J Alzheimers Dis.

[75] Graves AB, Bowen JD, Rajaram L, McCormick WC, McCurry SM, Schellenberg GD, Larson EB (1999). Impaired olfaction as a marker for cognitive decline: interaction with apolipoprotein E epsilon4 status. Neurology.

[76] Olofsson JK, Larsson M, Roa C, Wilson DA, Jonsson Laukka E. Interaction between odor identification deficit and APOE4 predicts 6-year cognitive decline in elderly individuals. Behav Genet. 2019. 10.1007/s10519-019-09980-9 PMC6941999 31760549

[77] Zhang J, Hao C, Jiang J, Feng Y, Chen X, Zheng Y, Liu J, Zhang Z, Long C, Yang L (2018). The mechanisms underlying olfactory deficits in apolipoprotein E-deficient mice: focus on olfactory epithelium and olfactory bulb. Neurobiol Aging.

[78] Murphy C (2019). Olfactory and other sensory impairments in Alzheimer disease. Nat Rev Neurol.

[79] Yoo HS, Jeon S, Chung SJ, Yun M, Lee PH, Sohn YH, Evans AC, Ye BS (2018). Olfactory dysfunction in Alzheimer's disease- and Lewy body-related cognitive impairment. Alzheimers Dement.

[80] Doty RL (2012). Olfactory dysfunction in Parkinson disease. Nat Rev Neurol.

[81] Ponsen MM, Stoffers D, Booij J, van Eck-Smit BL, Wolters E, Berendse HW (2004). Idiopathic hyposmia as a preclinical sign of Parkinson's disease. Ann Neurol.

[82] Ross GW, Petrovitch H, Abbott RD, Tanner CM, Popper J, Masaki K, Launer L, White LR (2008). Association of olfactory dysfunction with risk for future Parkinson's disease. Ann Neurol.

[83] Baba T, Kikuchi A, Hirayama K, Nishio Y, Hosokai Y, Kanno S, Hasegawa T, Sugeno N, Konno M, Suzuki K (2012). Severe olfactory dysfunction is a prodromal symptom of dementia associated with Parkinson's disease: a 3 year longitudinal study. Brain.

[84] Morley JF, Cohen A, Silveira-Moriyama L, Lees AJ, Williams DR, Katzenschlager R, Hawkes C, Shtraks JP, Weintraub D, Doty RL, Duda JE (2018). Optimizing olfactory testing for the diagnosis of Parkinson's disease: item analysis of the university of Pennsylvania smell identification test. NPJ Parkinsons Dis.

[85] Duyckaerts C, Delatour B, Potier MC (2009). Classification and basic pathology of Alzheimer disease. Acta Neuropathol.

[86] Pearson RC, Esiri MM, Hiorns RW, Wilcock GK, Powell TP (1985). Anatomical correlates of the distribution of the pathological changes in the neocortex in Alzheimer disease. Proc Natl Acad Sci U S A.

[87] Mrdjen D, Fox EJ, Bukhari SA, Montine KS, Bendall SC, Montine TJ (2019). The basis of cellular and regional vulnerability in Alzheimer's disease. Acta Neuropathol.

[88] Braak H, Braak E (1991). Neuropathological stageing of Alzheimer-related changes. Acta Neuropathol.

[89] Braak H, Braak E, Bohl J, Bratzke H (1998). Evolution of Alzheimer's disease related cortical lesions. J Neural Transm Suppl.

[90] Braak H, Del Tredici K (2015). The preclinical phase of the pathological process underlying sporadic Alzheimer's disease. Brain.

[91] Esiri MM, Wilcock GK (1984). The olfactory bulbs in Alzheimer's disease. J Neurol Neurosurg Psychiatry.

[92] Struble RG, Clark HB (1992). Olfactory bulb lesions in Alzheimer's disease. Neurobiol Aging.

[93] ter Laak HJ, Renkawek K, van Workum FP (1994). The olfactory bulb in Alzheimer disease: a morphologic study of neuron loss, tangles, and senile plaques in relation to olfaction. Alzheimer Dis Assoc Disord.

[94] Kovacs T, Cairns NJ (1999). Lantos PL: beta-amyloid deposition and neurofibrillary tangle formation in the olfactory bulb in ageing and Alzheimer's disease. Neuropathol Appl Neurobiol.

[95] Kovacs T, Cairns NJ, Lantos PL (2001). Olfactory centres in Alzheimer's disease: olfactory bulb is involved in early Braak's stages. Neuroreport.

[96] Tsuboi Y, Wszolek ZK, Graff-Radford NR, Cookson N, Dickson DW (2003). Tau pathology in the olfactory bulb correlates with Braak stage, Lewy body pathology and apolipoprotein epsilon4. Neuropathol Appl Neurobiol.

[97] Jellinger KA, Attems J (2005). Alzheimer pathology in the olfactory bulb. Neuropathol Appl Neurobiol.

[98] Attems J, Jellinger KA (2006). Olfactory tau pathology in Alzheimer disease and mild cognitive impairment. Clin Neuropathol.

[99] Saiz-Sanchez D, De la Rosa-Prieto C, Ubeda-Banon I, Martinez-Marcos A (2015). Interneurons, tau and amyloid-beta in the piriform cortex in Alzheimer's disease. Brain Struct Funct.

[100] Saiz-Sanchez D, Flores-Cuadrado A, Ubeda-Banon I, de la Rosa-Prieto C, Martinez-Marcos A (2016). Interneurons in the human olfactory system in Alzheimer's disease. Exp Neurol.

[101] Del Tredici K, Rub U, De Vos RA, Bohl JR, Braak H (2002). Where does parkinson disease pathology begin in the brain?. J Neuropathol Exp Neurol.

[102] Braak H, Muller CM, Rub U, Ackermann H, Bratzke H, de Vos RA, Del Tredici K. Pathology associated with sporadic Parkinson's disease--where does it end? J Neural Transm Suppl. 2006:89–97. 10.1007/978-3-211-45295-0_15 17017514

[103] Daniel SE, Hawkes CH (1992). Preliminary diagnosis of Parkinson's disease by olfactory bulb pathology. Lancet.

[104] Hoogland PV, van den Berg R, Huisman E (2003). Misrouted olfactory fibres and ectopic olfactory glomeruli in normal humans and in Parkinson and Alzheimer patients. Neuropathol Appl Neurobiol.

[105] Hubbard PS, Esiri MM, Reading M, McShane R, Nagy Z (2007). Alpha-synuclein pathology in the olfactory pathways of dementia patients. J Anat.

[106] Silveira-Moriyama L, Holton JL, Kingsbury A, Ayling H, Petrie A, Sterlacci W, Poewe W, Maier H, Lees AJ, Revesz T (2009). Regional differences in the severity of Lewy body pathology across the olfactory cortex. Neurosci Lett.

[107] Beach TG, White CL, Hladik CL, Sabbagh MN, Connor DJ, Shill HA, Sue LI, Sasse J, Bachalakuri J, Henry-Watson J (2009). Olfactory bulb alpha-synucleinopathy has high specificity and sensitivity for Lewy body disorders. Acta Neuropathol.

[108] Jellinger KA (2009). Olfactory bulb alpha-synucleinopathy has high specificity and sensitivity for Lewy body disorders. Acta Neuropathol.

[109] Parkkinen L, Silveira-Moriyama L, Holton JL, Lees AJ, Revesz T (2009). Can olfactory bulb biopsy be justified for the diagnosis of Parkinson's disease? Comments on "olfactory bulb alpha-synucleinopathy has high specificity and sensitivity for Lewy body disorders". Acta Neuropathol.

[110] Tabaton M, Monaco S, Cordone MP, Colucci M, Giaccone G, Tagliavini F, Zanusso G (2004). Prion deposition in olfactory biopsy of sporadic Creutzfeldt-Jakob disease. Ann Neurol.

[111] Duda JE (2010). Olfactory system pathology as a model of Lewy neurodegenerative disease. J Neurol Sci.

[112] Kesslak JP, Nalcioglu O, Cotman CW (1991). Quantification of magnetic resonance scans for hippocampal and parahippocampal atrophy in Alzheimer's disease. Neurology.

[113] Di Paola M, Macaluso E, Carlesimo GA, Tomaiuolo F, Worsley KJ, Fadda L, Caltagirone C (2007). Episodic memory impairment in patients with Alzheimer's disease is correlated with entorhinal cortex atrophy. A voxel-based morphometry study. J Neurol.

[114] Prestia A, Drago V, Rasser PE, Bonetti M, Thompson PM, Frisoni GB (2010). Cortical changes in incipient Alzheimer's disease. J Alzheimers Dis.

[115] Imabayashi E, Matsuda H, Tabira T, Arima K, Araki N, Ishii K, Yamashita F, Iwatsubo T (2013). Japanese Alzheimer's disease neuroimaging I: comparison between brain CT and MRI for voxel-based morphometry of Alzheimer's disease. Brain Behav.

[116] Vasavada MM, Wang J, Eslinger PJ, Gill DJ, Sun X, Karunanayaka P, Yang QX (2015). Olfactory cortex degeneration in Alzheimer's disease and mild cognitive impairment. J Alzheimers Dis.

[117] Thomann PA, Dos Santos V, Toro P, Schonknecht P, Essig M, Schroder J (2009). Reduced olfactory bulb and tract volume in early Alzheimer's disease--a MRI study. Neurobiol Aging.

[118] Thomann PA, Dos Santos V, Seidl U, Toro P, Essig M, Schroder J (2009). MRI-derived atrophy of the olfactory bulb and tract in mild cognitive impairment and Alzheimer's disease. J Alzheimers Dis.

[119] Servello A, Fioretti A, Gualdi G, Di Biasi C, Pittalis A, Sollaku S, Pavaci S, Tortorella F, Fusetti M, Valenti M (2015). Olfactory dysfunction, olfactory bulb volume and Alzheimer's disease: is there a correlation? A pilot Study1. J Alzheimers Dis.

[120] Vogels OJ (1994). Alzheimer's disease: stereology in search of specific markers. Neurobiol Aging.

[121] West MJ, Coleman PD, Flood DG, Troncoso JC (1994). Differences in the pattern of hippocampal neuronal loss in normal ageing and Alzheimer's disease. Lancet.

[122] Gomez-Isla T, Price JL, McKeel DW, Morris JC, Growdon JH, Hyman BT (1996). Profound loss of layer II entorhinal cortex neurons occurs in very mild Alzheimer's disease. J Neurosci.

[123] Price JL, Ko AI, Wade MJ, Tsou SK, McKeel DW, Morris JC (2001). Neuron number in the entorhinal cortex and CA1 in preclinical Alzheimer disease. Arch Neurol.

[124] West MJ, Kawas CH, Stewart WF, Rudow GL, Troncoso JC (2004). Hippocampal neurons in pre-clinical Alzheimer's disease. Neurobiol Aging.

[125] Kril JJ, Hodges J, Halliday G (2004). Relationship between hippocampal volume and CA1 neuron loss in brains of humans with and without Alzheimer's disease. Neurosci Lett.

[126] von Gunten A, Kovari E, Rivara CB, Bouras C, Hof PR, Giannakopoulos P (2005). Stereologic analysis of hippocampal Alzheimer's disease pathology in the oldest-old: evidence for sparing of the entorhinal cortex and CA1 field. Exp Neurol.

[127] von Gunten A, Kovari E, Bussiere T, Rivara CB, Gold G, Bouras C, Hof PR, Giannakopoulos P (2006). Cognitive impact of neuronal pathology in the entorhinal cortex and CA1 field in Alzheimer's disease. Neurobiol Aging.

[128] Giannakopoulos P, von Gunten A, Kovari E, Gold G, Herrmann FR, Hof PR, Bouras C (2007). Stereological analysis of neuropil threads in the hippocampal formation: relationships with Alzheimer's disease neuronal pathology and cognition. Neuropathol Appl Neurobiol.

[129] Mundinano IC, Caballero MC, Ordonez C, Hernandez M, DiCaudo C, Marcilla I, Erro ME, Tunon MT, Luquin MR (2011). Increased dopaminergic cells and protein aggregates in the olfactory bulb of patients with neurodegenerative disorders. Acta Neuropathol.

[130] Wattendorf E, Welge-Lussen A, Fiedler K, Bilecen D, Wolfensberger M, Fuhr P, Hummel T, Westermann B (2009). Olfactory impairment predicts brain atrophy in Parkinson's disease. J Neurosci.

[131] Wu X, Yu C, Fan F, Zhang K, Zhu C, Wu T, Li K, Chan P (2011). Correlation between progressive changes in piriform cortex and olfactory performance in early Parkinson's disease. Eur Neurol.

[132] Lee JE, Cho KH, Ham JH, Song SK, Sohn YH, Lee PH (2014). Olfactory performance acts as a cognitive reserve in non-demented patients with Parkinson's disease. Parkinsonism Relat Disord.

[133] Lee EY, Eslinger PJ, Du G, Kong L, Lewis MM, Huang X (2014). Olfactory-related cortical atrophy is associated with olfactory dysfunction in Parkinson's disease. Mov Disord.

[134] Ille R, Wabnegger A, Schwingenschuh P, Katschnig-Winter P, Kogl-Wallner M, Wenzel K, Schienle A (2015). Role of disgust proneness in Parkinson's disease: a voxel-based morphometry study. J Int Neuropsychol Soc.

[135] Eriksen N, Stark AK, Pakkenberg B. Age and Parkinson's disease-related neuronal death in the substantia nigra pars compacta. J Neural Transm Suppl. 2009:203–13. 10.1007/978-3-211-92660-4_16 20411779

[136] Stark AK, Pakkenberg B (2004). Histological changes of the dopaminergic nigrostriatal system in aging. Cell Tissue Res.

[137] Seidel K, Mahlke J, Siswanto S, Kruger R, Heinsen H, Auburger G, Bouzrou M, Grinberg LT, Wicht H, Korf HW (2015). The brainstem pathologies of Parkinson's disease and dementia with Lewy bodies. Brain Pathol.

[138] Jellinger KA (2012). Neuropathology of sporadic Parkinson's disease: evaluation and changes of concepts. Mov Disord.

[139] Lees AJ, Hardy J, Revesz T (2009). Parkinson's disease. Lancet.

[140] Iacono D, Geraci-Erck M, Rabin ML, Adler CH, Serrano G, Beach TG, Kurlan R (2015). Parkinson disease and incidental Lewy body disease: just a question of time?. Neurology.

[141] Chu Y, Buchman AS, Olanow CW, Kordower JH (2018). Do subjects with minimal motor features have prodromal Parkinson disease?. Ann Neurol.

[142] Pedersen KM, Marner L, Pakkenberg H, Pakkenberg B (2005). No global loss of neocortical neurons in Parkinson's disease: a quantitative stereological study. Mov Disord.

[143] Harding AJ, Stimson E, Henderson JM, Halliday GM (2002). Clinical correlates of selective pathology in the amygdala of patients with Parkinson's disease. Brain.

[144] Flores-Cuadrado A, Ubeda-Banon I, Saiz-Sanchez D, de la Rosa-Prieto C, Martinez-Marcos A (2016). Hippocampal alpha-synuclein and interneurons in Parkinson's disease: data from human and mouse models. Mov Disord.

[145] Joelving FC, Billeskov R, Christensen JR, West M, Pakkenberg B (2006). Hippocampal neuron and glial cell numbers in Parkinson's disease--a stereological study. Hippocampus.

[146] Pearce RK, Hawkes CH, Daniel SE (1995). The anterior olfactory nucleus in Parkinson's disease. Mov Disord.

[147] Huisman E, Uylings HB, Hoogland PV (2004). A 100% increase of dopaminergic cells in the olfactory bulb may explain hyposmia in Parkinson's disease. Mov Disord.

[148] Huisman E, Uylings HB, Hoogland PV (2008). Gender-related changes in increase of dopaminergic neurons in the olfactory bulb of Parkinson's disease patients. Mov Disord.

[149] Olender T, Keydar I, Pinto JM, Tatarskyy P, Alkelai A, Chien MS, Fishilevich S, Restrepo D, Matsunami H, Gilad Y, Lancet D (2016). The human olfactory transcriptome. BMC Genomics.

[150] Fernandez-Irigoyen J, Corrales FJ, Santamaria E (2012). Proteomic atlas of the human olfactory bulb. J Proteome.

[151] Lachen-Montes M, Gonzalez-Morales A, Schvartz D, Zelaya MV, Ausin K, Fernandez-Irigoyen J, Sanchez JC, Santamaria E (2019). The olfactory bulb proteotype differs across frontotemporal dementia spectrum. J Proteome.

[152] Fernandez-Irigoyen J, Santamaria E (2019). Olfactory proteotyping: towards the enlightenment of the neurodegeneration. Neural Regen Res.

[153] Palomino-Alonso M, Lachen-Montes M, Gonzalez-Morales A, Ausin K, Perez-Mediavilla A, Fernandez-Irigoyen J, Santamaria E. Network-driven Proteogenomics unveils an aging-related imbalance in the olfactory IkappaBalpha-NFkappaB p65 complex functionality in Tg2576 Alzheimer's disease mouse model. Int J Mol Sci. 2017;18. 10.3390/ijms18112260 PMC5713230 29077059

[154] Lachen-Montes M, Gonzalez-Morales A, Palomino M, Ausin K, Gomez-Ochoa M, Zelaya MV, Ferrer I, Perez-Mediavilla A, Fernandez-Irigoyen J, Santamaria E (2019). Early-onset molecular derangements in the olfactory bulb of Tg2576 mice: novel insights into the stress-responsive olfactory kinase dynamics in Alzheimer's disease. Front Aging Neurosci.

[155] Zelaya MV, Perez-Valderrama E, de Morentin XM, Tunon T, Ferrer I, Luquin MR, Fernandez-Irigoyen J, Santamaria E (2015). Olfactory bulb proteome dynamics during the progression of sporadic Alzheimer's disease: identification of common and distinct olfactory targets across Alzheimer-related co-pathologies. Oncotarget.

[156] Lachen-Montes M, Gonzalez-Morales A, Zelaya MV, Perez-Valderrama E, Ausin K, Ferrer I, Fernandez-Irigoyen J, Santamaria E (2017). Olfactory bulb neuroproteomics reveals a chronological perturbation of survival routes and a disruption of prohibitin complex during Alzheimer's disease progression. Sci Rep.

[157] Lachen-Montes M, Zelaya MV, Segura V, Fernandez-Irigoyen J, Santamaria E (2017). Progressive modulation of the human olfactory bulb transcriptome during Alzheimer s disease evolution: novel insights into the olfactory signaling across proteinopathies. Oncotarget.

[158] Lachen-Montes M, Gonzalez-Morales A, Iloro I, Elortza F, Ferrer I, Gveric D, Fernandez-Irigoyen J, Santamaria E (2019). Unveiling the olfactory proteostatic disarrangement in Parkinson's disease by proteome-wide profiling. Neurobiol Aging.

[159] Lachen-Montes M, Gonzalez-Morales A, Fernandez-Irigoyen J, Santamaria E (2019). Deployment of label-free quantitative olfactory proteomics to detect cerebrospinal fluid biomarker candidates in Synucleinopathies. Methods Mol Biol.

[160] Tapia-Rojas C, Cabezas-Opazo F, Deaton CA, Vergara EH, Johnson GVW, Quintanilla RA (2019). It's all about tau. Prog Neurobiol.

[161] Riederer P, Berg D, Casadei N, Cheng F, Classen J, Dresel C, Jost W, Kruger R, Muller T (2019). Reichmann H, et al: alpha-Synuclein in Parkinson's disease: causal or bystander?. J Neural Transm (Vienna).

[162] Walker LC (2018). Prion-like mechanisms in Alzheimer disease. Handb Clin Neurol.

[163] Angot E, Steiner JA, Hansen C, Li JY, Brundin P (2010). Are synucleinopathies prion-like disorders?. Lancet Neurol.

[164] Dunning CJ, Reyes JF, Steiner JA, Brundin P (2012). Can Parkinson's disease pathology be propagated from one neuron to another?. Prog Neurobiol.

[165] Olanow CW, Brundin P (2013). Parkinson's disease and alpha synuclein: is Parkinson's disease a prion-like disorder?. Mov Disord.

[166] George S, Rey NL, Reichenbach N, Steiner JA (2013). Brundin P: alpha-Synuclein: the long distance runner. Brain Pathol.

[167] Prymaczok NC, Riek R, Gerez J (2016). More than a rumor spreads in Parkinson's disease. Front Hum Neurosci.

[168] McCann H, Cartwright H, Halliday GM (2016). Neuropathology of alpha-synuclein propagation and braak hypothesis. Mov Disord.

[169] Steiner JA, Quansah E, Brundin P (2018). The concept of alpha-synuclein as a prion-like protein: ten years after. Cell Tissue Res.

[170] Karpowicz RJ, Trojanowski JQ, Lee VM (2019). Transmission of alpha-synuclein seeds in neurodegenerative disease: recent developments. Lab Investig.

[171] Hansen C, Li JY (2012). Beyond alpha-synuclein transfer: pathology propagation in Parkinson's disease. Trends Mol Med.

[172] Makin S (2016). Pathology: the prion principle. Nature.

[173] Brundin P, Melki R (2017). Prying into the prion hypothesis for Parkinson's disease. J Neurosci.

[174] Surmeier DJ, Obeso JA, Halliday GM (2017). Parkinson's disease is not simply a prion disorder. J Neurosci.

[175] Braak H, Del Tredici K, Bratzke H, Hamm-Clement J, Sandmann-Keil D, Rub U (2002). Staging of the intracerebral inclusion body pathology associated with idiopathic Parkinson's disease (preclinical and clinical stages). J Neurol.

[176] Braak H, Del Tredici K. Potential pathways of abnormal tau and alpha-Synuclein dissemination in sporadic Alzheimer's and Parkinson's diseases. Cold Spring Harb Perspect Biol. 2016;8. 10.1101/cshperspect.a023630 PMC5088528 27580631

[177] Braak H, Del Tredici K (2011). Alzheimer's pathogenesis: is there neuron-to-neuron propagation?. Acta Neuropathol.

[178] Braak H, Rub U, Gai WP, Del Tredici K (2003). Idiopathic Parkinson's disease: possible routes by which vulnerable neuronal types may be subject to neuroinvasion by an unknown pathogen. J Neural Transm (Vienna).

[179] Del Tredici K, Braak H (2012). Lewy pathology and neurodegeneration in premotor Parkinson's disease. Mov Disord.

[180] Doty RL (2008). The olfactory vector hypothesis of neurodegenerative disease: is it viable?. Ann Neurol.

[181] Hawkes CH, Del Tredici K, Braak H (2007). Parkinson's disease: a dual-hit hypothesis. Neuropathol Appl Neurobiol.

[182] Hawkes CH, Del Tredici K, Braak H (2009). Parkinson's disease: the dual hit theory revisited. Ann N Y Acad Sci.

[183] Borghammer P (2018). How does parkinson's disease begin? Perspectives on neuroanatomical pathways, prions, and histology. Mov Disord.

[184] Lionnet A, Leclair-Visonneau L, Neunlist M, Murayama S, Takao M, Adler CH, Derkinderen P, Beach TG (2018). Does Parkinson's disease start in the gut?. Acta Neuropathol.

[185] Dujardin S, Lecolle K, Caillierez R, Begard S, Zommer N, Lachaud C, Carrier S, Dufour N, Auregan G, Winderickx J (2014). Neuron-to-neuron wild-type tau protein transfer through a trans-synaptic mechanism: relevance to sporadic tauopathies. Acta Neuropathol Commun.

[186] Villar-Pique A, Lopes da Fonseca T, Sant’Anna R, Szego EM, Fonseca-Ornelas L, Pinho R, Carija A, Gerhardt E, Masaracchia C, Abad Gonzalez E (2016). Environmental and genetic factors support the dissociation between alpha-synuclein aggregation and toxicity. Proc Natl Acad Sci U S A.

[187] Cavaliere F, Cerf L, Dehay B, Ramos-Gonzalez P, De Giorgi F, Bourdenx M, Bessede A, Obeso JA, Matute C, Ichas F, Bezard E (2017). In vitro alpha-synuclein neurotoxicity and spreading among neurons and astrocytes using Lewy body extracts from Parkinson disease brains. Neurobiol Dis.

[188] Rey NL, George S, Steiner JA, Madaj Z, Luk KC, Trojanowski JQ, Lee VM, Brundin P (2018). Spread of aggregates after olfactory bulb injection of alpha-synuclein fibrils is associated with early neuronal loss and is reduced long term. Acta Neuropathol.

[189] Palop JJ, Chin J, Mucke L (2006). A network dysfunction perspective on neurodegenerative diseases. Nature.

[190] Mikkonen M, Alafuzoff I, Tapiola T, Soininen H, Miettinen R (1999). Subfield- and layer-specific changes in parvalbumin, calretinin and calbindin-D28K immunoreactivity in the entorhinal cortex in Alzheimer's disease. Neuroscience.

[191] Saiz-Sanchez D, Ubeda-Banon I, Flores-Cuadrado A, Gonzalez-Rodriguez M, Villar-Conde S, Astillero-Lopez V, Martinez-Marcos A. Somatostatin, olfaction and neurodegeneration. Front Neurosci. 2020;14:96. 10.3389/fnins.2020.00096 PMC7042373 32140092

[192] Sporns O, Tononi G, Kotter R (2005). The human connectome: a structural description of the human brain. PLoS Comput Biol.

[193] Fox MD (2018). Mapping symptoms to brain networks with the human Connectome. N Engl J Med.

[194] Mears D, Pollard HB (2016). Network science and the human brain: using graph theory to understand the brain and one of its hubs, the amygdala, in health and disease. J Neurosci Res.

[195] Crossley NA, Mechelli A, Scott J, Carletti F, Fox PT, McGuire P, Bullmore ET (2014). The hubs of the human connectome are generally implicated in the anatomy of brain disorders. Brain.

[196] Rees CL, Wheeler DW, Hamilton DJ, White CM, Komendantov AO, Ascoli GA. Graph theoretic and motif analyses of the hippocampal neuron type potential Connectome. eNeuro. 2016;3. 10.1523/ENEURO.0205-16.2016 PMC5114701 27896314

[197] Jagust W (2018). Imaging the evolution and pathophysiology of Alzheimer disease. Nat Rev Neurosci.

[198] Chen XQ, Mobley WC (2019). Alzheimer disease pathogenesis: insights from molecular and cellular biology studies of Oligomeric Abeta and tau species. Front Neurosci.

[199] Nelson PT, Abner EL, Patel E, Anderson S, Wilcock DM, Kryscio RJ, Van Eldik LJ, Jicha GA, Gal Z, Nelson RS (2018). The amygdala as a locus of pathologic Misfolding in neurodegenerative diseases. J Neuropathol Exp Neurol.

[200] Braak H, Del Tredici K (2017). Neuropathological staging of brain pathology in sporadic Parkinson's disease: separating the wheat from the chaff. J Park Dis.

[201] Flores-Cuadrado A, Ubeda-Banon I, Saiz-Sanchez D (2017). Martinez-Marcos a: alpha-Synucleinopathy in the human amygdala in Parkinson disease: differential vulnerability of Somatostatin- and Parvalbumin-expressing neurons. J Neuropathol Exp Neurol.

[202] Rey NL, Wesson DW, Brundin P (2018). The olfactory bulb as the entry site for prion-like propagation in neurodegenerative diseases. Neurobiol Dis.

